# Synthesis and crystal structures of a bis­(3-hy­droxy-cyclo­hex-2-en-1-one) and two hexa­hydro­quinoline derivatives

**DOI:** 10.1107/S2056989019017018

**Published:** 2020-01-03

**Authors:** Scott A. Steiger, Chun Li, Christina Gates, Nicholas R. Natale

**Affiliations:** a Department of Biomedical and Pharmaceutical Sciences, The University of Montana, 32 Campus Drive, Missoula, MT 59812, USA; b Department of Chemistry, Ithaca College, 953 Danby Road, Ithaca, NY 14850, USA

**Keywords:** crystal structure, hexa­hydro­quinoline, hydrogen bonding

## Abstract

The title compound **I**, 2,2′-[(2-nitro­phen­yl)methyl­ene]bis­(3-hy­droxy-5,5-di­methyl­cyclo­hex-2-enone), C_23_H_27_NO_6_, features a 1,3-ketone–enol conformation which is stabilized by two intra­molecular hydrogen bonds. The most prominent inter­molecular inter­actions in compound **I** are C—H⋯O hydrogen bonds, which link mol­ecules into a two-dimensional network parallel to the (001) plane and a chain perpendicular to (11). Both title compounds **II**, ethyl 4-(4-hy­droxy-3,5-di­meth­oxy­phen­yl)-2,7,7-trimethyl-5-oxo-1,4,5,6,7,8-hexa­hydro­quinoline-3-carb­oxyl­ate, C_23_H_29_NO_6_, and **III**, ethyl 4-(anthracen-9-yl)-2,7,7-trimethyl-5-oxo-1,4,5,6,7,8-hexa­hydro­quinoline-3-carboxyl­ate, C_29_H_29_NO_3_, share the same structural features, such as a shallow boat conformation of the di­hydro­pyridine group and an orthogonal aryl group attached to the di­hydro­pyridine. Inter­molecular N—H⋯O bonding is present in the crystal packing of both compound **II** and **III**.

## Abstract 2

The syntheses and crystal structures of three compounds, 2,2′-[(2-nitro­phen­yl)methyl­ene]bis­(3-hy­droxy-5,5-di­methyl­cyclo­hex-2-enone) (**I**), ethyl 4-(4-hy­droxy-3,5-di­meth­oxy­phen­yl)-2,7,7-trimethyl-5-oxo-1,4,5,6,7,8-hexa­hydro­quinoline-3-carboxyl­ate (**II**), and ethyl 4-(anthracen-9-yl)-2,7,7-trimethyl-5-oxo-1,4,5,6,7,8-hexa­hydro­quinoline-3-carboxyl­ate (**III**), are reported.

## Chemical context   

4-Aryl-1,4-di­hydro­pyridines (DHPs) that bind the L-type voltage-gated calcium channels (VGCC) have been applied in general medical practice for over three decades. (Zamponi, 2016 ▸). Many modifications on 1,4-DHP have been performed to obtain active compounds such as calcium-channel agonists or antagonists. (Martín *et al.*, 1995 ▸; Rose, 1990 ▸; Rose & Dräger, 1992 ▸; Trippier *et al.* 2013 ▸) One such modification is fusing a cyclo­hexa­none ring to form hexa­hydro­quinolone (HHQ), in which the orientation of the carbonyl group of the ester substituent at the 5-position in the 1,4-DHP ring has been fixed. This class of compounds has been shown to have calcium-channel antagonistic activity (Aygün Cevher *et al.*, 2019 ▸), inhibit the multidrug-resistance transporter (MDR) (Shahraki *et al.*, 2017 ▸), as well as possess anti-inflammatory and stem-cell differentiation properties, and has been implicated in slowing neurodegenerative disorders. (Trippier *et al.*, 2013 ▸). In the HHQ literature, specific substitution of the cyclo­hexenone ring can confer sub-type selectivity at the voltage-gated calcium channel (Schaller *et al.*, 2018 ▸). Our group has been inter­ested in bioisosteric 4-isoxazolyl-di­hydro­pyridines at the VGCC (Schauer *et al.*, 1986 ▸; Zamponi *et al.*, 2003 ▸; Natale *et al.*, 2014 ▸) and MDR (Steiger *et al.*, 2017 ▸), and continue our studies towards understanding stereoelectronic effects, which define selectivity, as well as to explore the scope and limitations of our synthetic methodologies (Steiger *et al.*, 2016 ▸). These inter­ests led us to continue our pursuit of crystallographic studies in this area (Steiger *et al.*, 2014*a* ▸,*b* ▸; 2018 ▸).
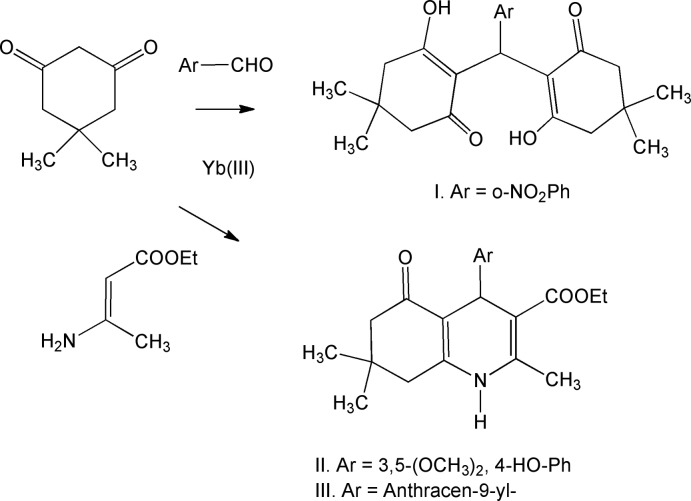


## Structural commentary   

**Figure 1:**
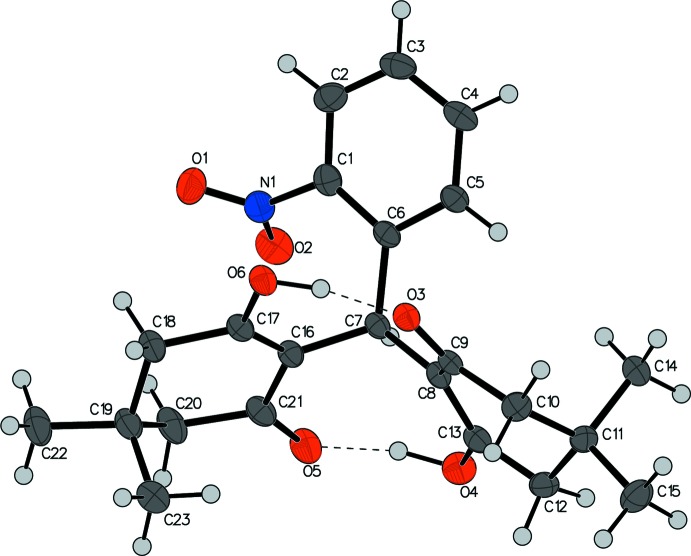
The asymmetric unit of compound **I** showing the atom-labeling scheme. Displacement ellipsoids are drawn at the 50% probability level. The dashed lines indicate intra­molecular O—H⋯O hydrogen bonds.

Compound **I** crystallizes in the triclinic *P* 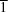 space group with one independent mol­ecule in the asymmetric unit (Fig. 1 ▸). As in other bis­(3-hy­droxy-5,5-di­methyl­cyclo­hex-2-enone) compounds, in compound **I** the 1,3-ketone–enol conformation is stabilized by two inter­nal hydrogen bonds between two pairs of enols and ketones that bridge the two hy­droxy­cyclo­hexenones, in addition to the bridging carbon C7. The two hy­droxy­cyclo­hexenones are arranged along a pseudo-mirror plane formed by atoms C15, C11, C8, C7, C16, C19, and C22, which has a root-mean-square deviation (RMSD) of 0.025 Å. The phenyl ring attached to C7 flaps to one side of the above plane, with a plane normal angle of 44.34 (4)°.

**Figure 2:**
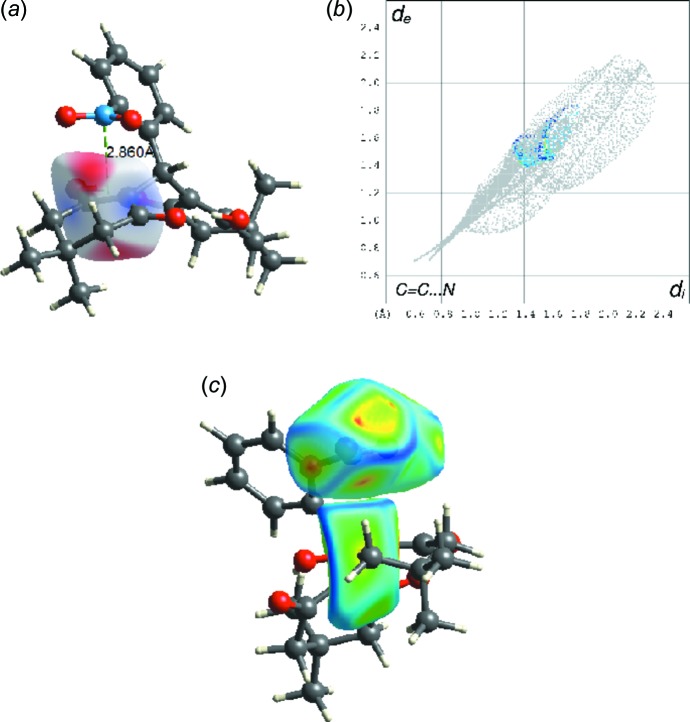
(*a*) View of the three-dimensional Hirshfeld surface of C16—C17 mapped over electrostatic potentials, over the range of −0.0221 to 0.9216 arbitrary units. (*b*) The two-dimensional fingerprint plot for the C=C⋯N inter­action. (*c*) The Hirshfeld surfaces of NO_2_ and C16—C17 mapped over curvedness.

Both 3-hy­droxy-5,5-dimethyl-cyclo­hex-2-en-1-one rings adopt an envelope conformation, with both methyl groups C14 and C23 having an axial orientation being trans to each other. As a result of the steric effect of the neighboring atoms and groups, instead of being on the same plane as the phenyl ring, the mean plane formed by the NO_2_ group is rotated out of the plane of the aromatic system with an angle of 52.85 (6)°. This may indicate a possible π–π inter­action between the NO_2_ group and the ketone–enol C=C bond, evidenced by a short-contact N1⋯C16 distance of 2.816 (2) Å and a short distance of 2.860 Å between N1 and the midpoint of the C16=C17 double bond. The inter­action of the NO_2_ group and the enol C16=C17 double bond were analyzed using Hirshfeld surface analysis and qu­anti­fied using the associated two-dimensional fingerprint plot (Fig. 2 ▸), both performed with *CrystalExplorer17.5* (Turner *et al.*, 2017 ▸). The electrostatic potentials were calculated using *TONTO* integrated within *CrystalExplorer*. Hirshfeld surfaces of the NO_2_ group and C16=C17 mapped over curvedness are shown in Fig. 2 ▸(c). The flat yellowish surfaces confirm that an intra­molecular π–π inter­action takes place between the NO_2_ group and the enol double bond. This is also evidence that the π-hole inter­action can stabilize conformers when the inter­acting atom is four or five bonds away from the N atom of a nitro aromatic compound (Franconetti *et al.*, 2019 ▸).

**Figure 3:**
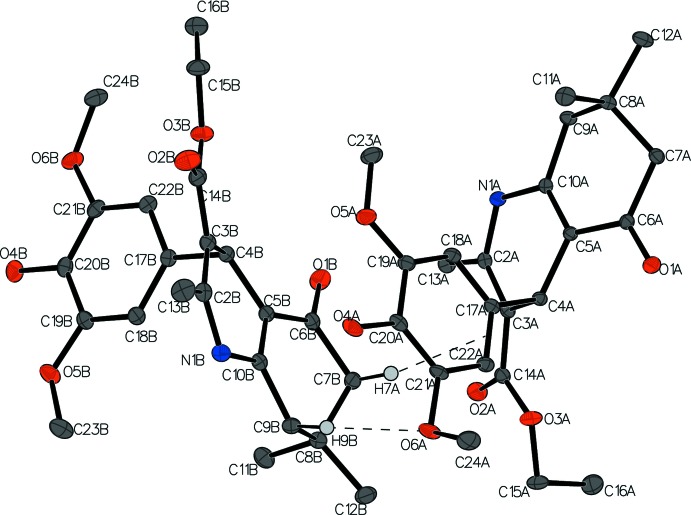
The asymmetric unit of compound **II** showing the atom-labeling scheme. Displacement ellipsoids are drawn at the 50% probability level. The dashed lines indicate the C9*B*—H9*B*⋯O6*A* hydrogen bond and the C—H⋯ π inter­action between H7*A* and the C17*A*—C22*A* bond. Other hydrogen atoms have been omitted for clarity.

Compounds **II** and **III** both crystallized racemically in the monoclinic space group *P*2_1_/*n*. The asymmetric unit of compound **II** contains two independent mol­ecules (*A* and *B*), both in the same enanti­omeric configuration. The overall unit cell is racemic with four pairs of racemates. Compound **III** has only one independent mol­ecule in the asymmetric unit. The displacement ellipsoid plots showing the atomic numbering of compounds **II** and **III** are presented in Figs. 3 ▸ and 4 ▸, respectively.

As in the other 4-aryl-hexa­hydro­quinoline derivatives (Steiger *et al.*, 2014*a* ▸,*b* ▸; 2018 ▸) that we have reported, compound **II** has a flattened boat conformation on the 1,4-DHP ring. The mean plane defined by atoms C2, C3, C5, and C10 is planar with an RMSD of 0.000 and 0.006 Å for *A* and *B*, respectively. Atoms N1 and C4 are displaced slightly from the mean plane at distances of 0.1696 (11) Å for N1*A* and 0.1867 (11) Å for N1*B*, and 0.3722 (13) Å for C4*A* and 0.3506 (13) Å for C4*B*, respectively. The 4-di­hydroxy­lmethoxyphenyl ring is almost orthogonal to the 1,4-DHP basal plane comprising atoms C2, C3, C5, and C10, making dihedral angles of 88.03 (3) and 81.05 (3)° in **II** ***A*** and **II** ***B***, respectively. The ring puckering parameters for the cyclo­hexa­none ring (C5–C10) indicate that it adopts an envelope conformation: *Q* = 0.4631 Å, θ = 58.01°, and φ = 168.1681° for **II** ***A*** and *Q* = 0.4592 Å, θ = 124.10°, and φ = 344.3794° for **II** ***B***.

In the mol­ecule of compound **II**, the orientations of the ethyl groups on the ester and of the meth­oxy groups on the phenyl rings are different in mol­ecules *A* and *B*. The hydroxyl and meth­oxy groups are mostly co-planar with the phenyl ring to which they are attached in both mol­ecules *A* and *B.* The exception is one of the methyl groups in mol­ecule *A*, C24*A*, which protrudes out of the phenyl plane with a displacement of 1.2802 (12) Å. The angle between the O6*A*—C24*A* bond and the normal to the phenyl plane is 154.38 (5)°. Similarly, the ethyl group on the ester group in mol­ecule *B* is co-planar with the ester atoms O2*B*, O3*B*, and C14*B* whereas in mol­ecule *A*, the ethyl group is folded with an angle of 14.94 (10)° between the C15*A*—C16*A* bond and the normal to the O2*A*/O3*A*/C14*A* plane with atom C16*A* displaced by 1.656 (3) Å from the plane. These orientations imply that these two functional groups are flexible in the structure.

Although compounds **II** and **III** share the same structural features, such as the envelope conformation of the cyclo­hexa­none ring and the pseudo-axial position of the 4-aryl group, they exhibit differences, especially in the conformation of the 1,4-DHP ring. In compound **III**, atoms N1 and C4 are only slightly displaced from C2/C3/C5/C10 mean plane at distances of 0.107 (2) and 0.092 (2) Å, respectively. There is a short contact of 1.88 Å between hydrogen atoms H4 and H27. A C—H⋯π contact of 2.47 Å also exists between C19—H19 and the C5—C10 bond.

In compound **III**, the anthracenyl group bis­ects the basal plane of the 1,4-DHP ring, with N1⋯C4—C17—C18 torsion angle of 2.09 (15)°. As a result of the elongated aromatic system, the ethyl group on the ester is stabilized in a folded position by a weak C—H⋯π inter­action between C16—H16*B* and C25–C30 ring, with an H16-to-plane distance of 2.82 Å. The O=C—O ester group is no longer co-planar with the 1,4-DHP basal plane and the O2—C14—C3—C2 torsion angle is −25.10 (19)°.

## Supra­molecular features   

**Figure 5:**
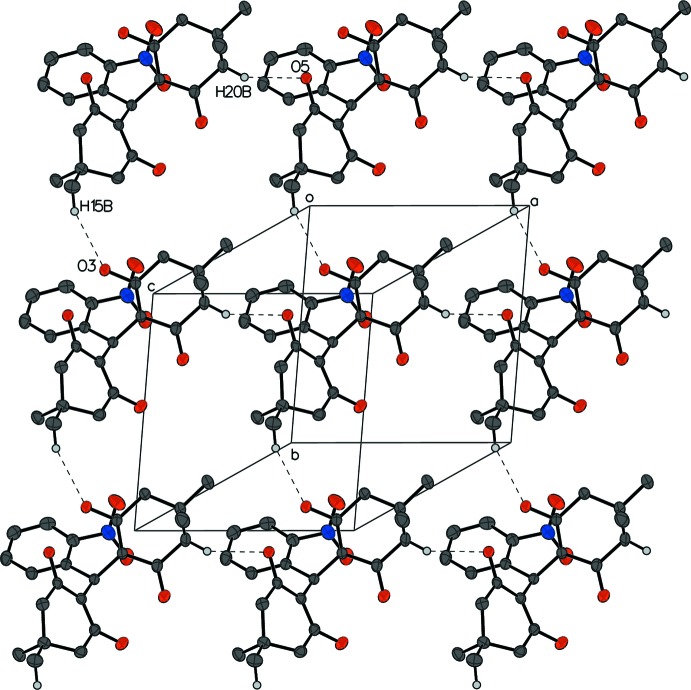
The packing of compound **I** showing the two-dimensional network parallel to the (001) plane. For clarity, H atoms not participating in hydrogen bonds are omitted, and participating atoms are labeled once.

**Figure 6:**
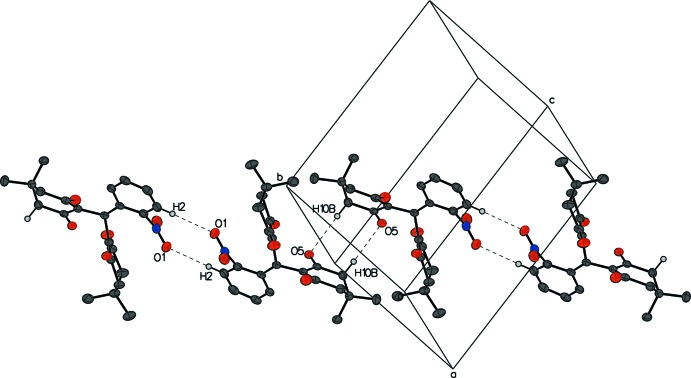
The packing of compound **I** showing a chain of alternating enanti­omers. For clarity, H atoms not participating in hydrogen bonds are omitted, and participating atoms are labeled once.

In compound **I**, C15—H15*B*⋯O3^i^ and C20—H20*B*⋯O5^ii^ and hydrogen bonds (Table 1 ▸) between the same enanti­omers form a two-dimensional network parallel to (001), with one chain running along the *a*-axis direction and the other along the *b*-axis direction (Fig. 5 ▸). Other inter­molecular O—H inter­actions such as C10—H10*B*⋯O5^ii^ and C2—H2⋯O1^i^ between a pair of enanti­omers form a chain of alternating enanti­omers (Fig. 6 ▸)

**Figure 7:**
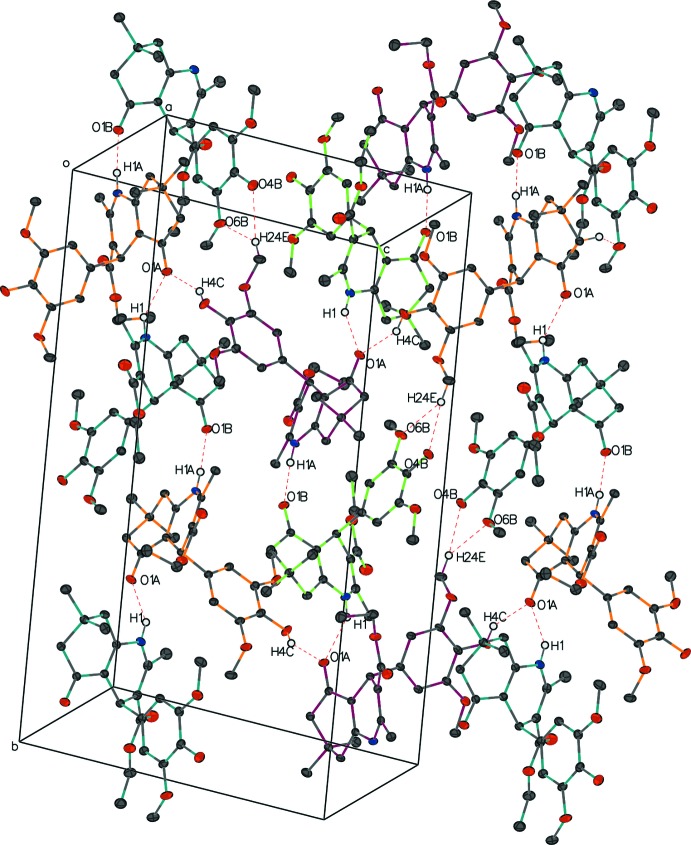
The packing of compound **II** showing an array of columns along the *b* axis formed by hydrogen bonds. Atoms involved in hydrogen bonds are labeled. H atoms not involved in hydrogen bonds are omitted for clarity. Mol­ecules *A* and *B* are colored in orange and lime, respectively. Mol­ecules colored in magenta are the enanti­omers of mol­ecule *A*, and those colored in teal are the enanti­omers of mol­ecule *B*.

**Figure 8:**
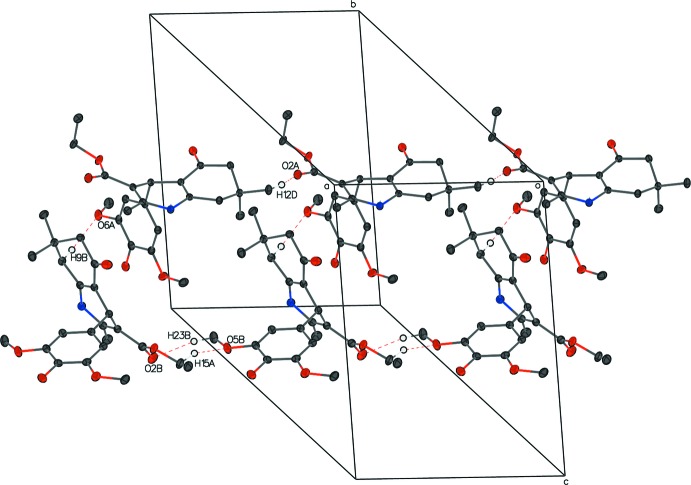
The packing of compound **II** showing the chains formed by *A* and *B* mol­ecules along the *a* axis. For clarity, H atoms not participating in hydrogen bonds are omitted, and participating atoms are labeled once.

In compound **II**, there is a C9*B*—H9*B*⋯O6*A* hydrogen bond between mol­ecules *A* and *B*, with distance of 2.59 Å and a C—H⋯ π inter­action between C7*B*—H7*A* and the C17*A*—C22*A* bond with a distance of 2.6715 (6) Å. Links alternating between the two independent mol­ecules form a column through hydrogen bonds N1*A*—H1*A*⋯O1*B* ^ii^ and N1*B*—H1⋯O1*A* ^i^, which run along the *b-*axis direction. This column branches out through the O4*A*—H4*C*⋯O1*A* ^i^ and C24*A*—H24*E*⋯O4*B* ^v^ hydrogen bonds to another parallel column, forming a sheet perpendicular to (101) (Fig. 7 ▸). Weak C23*B*—H23*B*⋯O2*B* ^vi^ and C15*B*—H15*A*⋯O5*B* ^iii^ inter­actions link the *B* mol­ecules into a chain along the *a*-axis direction. A similar chain of *A* mol­ecules is formed through weak C12*A*—H12*D*⋯O2*A*′ inter­actions (Fig. 8 ▸). Other hydrogen bonds are listed in the Table 2 ▸.

**Figure 9:**
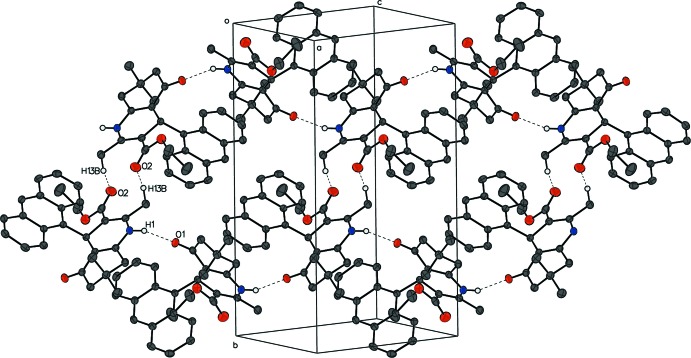
The packing of compound **III**. Cross-linked zigzag chains of alternating enanti­omers form a sheet. For clarity, H atoms not participating in hydrogen bonds are omitted, and participating atoms are labeled once.

In compound **III**, an N1—H1⋯O1^i^ hydrogen bond (Table 3 ▸) alternating between two enanti­omers results in a zigzag chain of racemic mol­ecules running perpendicular to the (101) plane. The C13—H13*B*⋯O2^ii^ hydrogen bond cross-links a pair of enanti­omers from different chains and forms a sheet of mol­ecules parallel to (10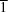) (Fig. 9 ▸). As a consequence of close packing, several short contacts are observed, *i.e*. an edge-to-edge π–π contact of 2.7636 (15) Å between C21 and C21^ii^, H4⋯C29^i^ = 2.76 Å and H7*A*⋯H24^i^ = 2.60 Å (symmetry codes as in Table 3 ▸).

## Database survey   

A search for aryl­bis­(3-hy­droxy-5,5-di­methyl­cyclo­hex-2-enone) compounds in the Cambridge Structural Database (CSD Version 5.40, update of August 2019; Groom *et al.*, 2016 ▸) gave 29 hits, among which are two NO_2_-phenyl­bis­(3-hy­droxy-5,5-di­methyl­cyclo­hex-2-enone) compounds. One is NO_2_ substituted at the *para* position (CSD refcode IRODID; Yao *et al.*, 2005 ▸) while the other is NO_2_ substituted at the *meta* position (VUZYIZ; Palakshi Reddy *et al.*, 2010 ▸) and both exhibit a similar structural configuration to that of compound **I**. However, with less steric effects surrounding the nitro group, both the *p*- and *m*-NO_2_ groups are tilted only slightly from the aromatic ring with torsion angles between the N=O and C=C bonds of *ca* 8.25 and 4.58°, respectively. In contrast, in compound **I** (an *o*-NO_2_ group), the torsion angle is 49.68 (6)°. The database search also found 20 4-aryl-hexa­hydro­quinoline-3-carboxyl­ate derivatives. All of them display the same common structural features as compounds **II** and **III** in this report, such as the flat-boat conformation of the 1,4-DHP ring, the envelope conformation of the fused cyclo­hexa­none ring, and the substituted phenyl ring at the pseudo-axial position and orthogonal to the 1,4-DHP ring.

## Synthesis and crystallization   

The synthesis was performed as outlined in the scheme. An oven-dried 100 ml round-bottom flask equipped with a magnetic stir bar was charged with 10 mmol of dimedone, 10 mmol of ethyl aceto­acetate and 5 mol% of ytterbium(III) tri­fluoro­methane­sulfonate (Wang *et al.*, 2005 ▸). The mixture was then taken up in 30 ml of absolute ethanol, capped and placed under an inert atmosphere of argon, after which the solution was allowed to stir at room temperature for 20 min. The appropriate corresponding benzaldehyde (10 mmol) and 10 mmol of ammonium acetate were added to the stirring solution, the solution was allowed to stir at room temperature for 48 h. Reaction progress was monitored *via* TLC. Once the reaction was complete, excess solvent was removed *via* rotary evaporation. The solution was then purified *via* silica column chromatography. The title compound was recrystallized by slow evaporation from hexane and ethyl acetate (*v*:*v* = 3:1).

2,2′-[(2-Nitro­phen­yl)methyl­ene]bis­(3-hy­droxy-5,5-di­meth­yl­cyclo­hex-2-enone) (**I**). ^1^H NMR (CDCl_3_) δ ppm 7.99 (*d*, *J* = 7.8 Hz, 1H); 7.39 (*ddd*, *J* = 1.37, 6.88 & 8 Hz, 1H); 7.35 (*dd*, *J* = 1.83 & 8.24 Hz, 1H); 7.30 (*ddd*, *J* = 1.37, 1.83 & 7.58 Hz, 1H), 5.01 (*s*, 1H); 3.35 (*s*, 1H); 2. 82 (*s*, 1H); 2.45 (*dd*, *J* = 4.35 & 14.76 Hz, 2H); 2.25 (*m*, 4H); 2.10 (*dd*, *J* = 1.83 & 14.20 Hz, 1 Hz); 2.04 (*d*, *J* =14.20 Hz, 1H); 1.14 (*s*, 3H); 1.11 (*s*, 3H); 1.04 (*s*, 3H); 0.95 (*s*, 3H). ^13^C NMR δ ppm 190.99, 189.51, 149.79, 132.16, 131.46, 129.67, 127.27, 124.44, 114.73, 46.93, 46.35, 32.00, 30.14, 28.62, 28.25. LC–MS calculated for C_23_H_27_NO_6_, observed *m*/*z* 414 ([*M*+1]^+^, 100% rel. intensity).

Ethyl 4-(4-hy­droxy-3,5-di­meth­oxy­phen­yl)-2,7,7-trimethyl-5-oxo-1,4,5,6,7,8-hexa­hydro­quinoline-3-carboxyl­ate (II). Spec­tra are similar to those for the product of the synthesis previously reported by Yang *et al.* (2011 ▸). ^1^H NMR (CDCl_3_) δ ppm 6.56 (*s*, 2H, Ar-H); 5.69 (*br.s*, 1H); 5.33 (*s*, 1H); 5.01 (*s*, 1H); 4.10 (*q*, 2H, *J* = 6Hz); 3.83 (*s*, 6H); 2.39 (*s*, 3H); 2.36, *s*, 1H); 2.225 (*q*, 2H, *J* = 16 Hz); 2.18 (*s*, 1H); 1.24 (*t*, 3H, *J* = 6Hz); 1.10(*s*, 3H); 0.99 (*s*, 3H). ^13^C NMR δ ppm 195.47, 167.42, 147.49, 146.49, 133.15, 112.33, 106.26, 104.98, 59.82, 56.23, 50.69, 36.34, 32.69, 29.58, 26.84, 19.53, 14.33. LC–MS calculated for C_23_H_29_NO_6_, observed *m*/*z* 831 ([*M*_2_+1]^+^, 100% rel. intensity), 416 ([*M*+1]^+^, 74), 262 ([*M*-4-Ar-H]^+^, 81).

Ethyl 4-(9′-Anthr­yl)-2,7,7-trimethyl-5-oxo-1,4,5,6,7,8-hexa­hydro­quinoline-3-carboxyl­ate (**III**). ^1^H NMR (CDCl_3_) δ ppm 9.09 (*d*, 1H); 8.29 (*s*, 2H); 7.93 (*m*, 2H); 7.57 (*m*, 1H); 7.43 (*m*, 1H); 7.33 (*m*, 2H); 6.68 (*s*, 1H); 5.92 (*br. s*, 1H); 3.7 (*m*, 2H, O*CH_2_*CH_3_), 2.06 (*d*, 1H, *J* = 16 Hz); 1.97 (*d*, 1H, *J* = 16 Hz); 0.5 (*t*, 3H, OCH_2_ *CH_3_, J* = 8 Hz). ^13^C NMR δppm 195.69, 167.41, 159.11, 147.37, 112.69, 111.72, 107.7, 59.39, 50.49, 32.27, 30.93, 29.11, 27.38, 19.11, 13.44. C_29_H_29_NO_3_, observed *m*/*z* 440 [*M*+1]^+^, 11.5% rel. intensity), 262 ([*M*-4-Ar-H]^+^, 100).

## Refinement   

Crystal data, data collection and structure refinement details are summarized in Table 4 ▸. Hydrogen atoms attached to carbon were placed in calculated positions (C—H = 0.95–0.98 A) and refined with isotropic displacement parameters 1.2–1.5 times those of the parent atoms. Hydrogen atoms attached to nitro­gen and oxygen were found in difference-Fourier maps and refined freely. In compound **III**, three reflections (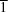
01, 110, and 020) affected by the beam stop were omitted because of poor agreement between the observed and calculated intensities.

## Supplementary Material

Crystal structure: contains datablock(s) I, II, III, global. DOI: 10.1107/S2056989019017018/dx2021sup1.cif

Structure factors: contains datablock(s) I. DOI: 10.1107/S2056989019017018/dx2021Isup2.hkl

Supporting information file. DOI: 10.1107/S2056989019017018/dx2021Isup5.cml

Structure factors: contains datablock(s) II. DOI: 10.1107/S2056989019017018/dx2021IIsup3.hkl

Supporting information file. DOI: 10.1107/S2056989019017018/dx2021IIsup6.cml

Structure factors: contains datablock(s) III. DOI: 10.1107/S2056989019017018/dx2021IIIsup4.hkl

CCDC references: 1973149, 1973148, 1973147

Additional supporting information:  crystallographic information; 3D view; checkCIF report

## Figures and Tables

**Figure 4:**
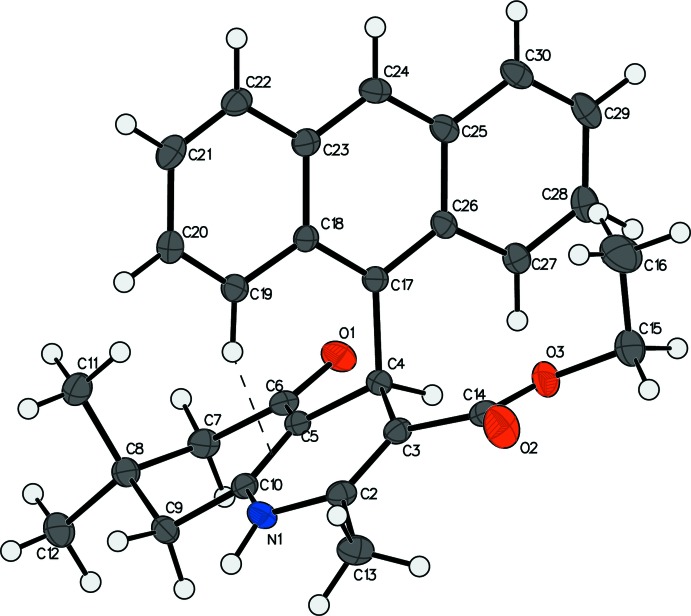
The asymmetric unit of compound **III** showing the atom-labeling scheme. Displacement ellipsoids are drawn at the 50% probability level. The C—H⋯π inter­action is indicated by a dashed line.

**Table 1:** Hydrogen-bond geometry (Å, °) for (I)

*D*—H⋯*A*	*D*—H	H⋯*A*	*D*⋯*A*	*D*—H⋯*A*
O3—H3⋯O5	0.97 (3)	1.62 (3)	2.5570 (16)	162 (3)
O6—H6⋯O4	1.08 (4)	1.58 (4)	2.6437 (19)	166 (3)
C2—H2⋯O1^i^	0.95	2.63	3.538 (2)	160
C10—H10*B*⋯O5^ii^	0.99	2.65	3.6138 (19)	165
C15—H15*B*⋯O3^iii^	0.98	2.58	3.505 (2)	157
C18—H18*B*⋯O1	1.04 (2)	2.67 (2)	3.381 (2)	125.5 (16)
C20—H20*B*⋯O5^iv^	0.99	2.43	3.332 (2)	152

Symmetry codes: (i) 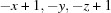; (ii) 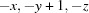; (iii) 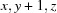; (iv) 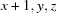.

**Table 2:** Hydrogen-bond geometry (Å, °) for (II)

*D*—H⋯*A*	*D*—H	H⋯*A*	*D*⋯*A*	*D*—H⋯*A*
O4*A*—H4*C*⋯O1*A* ^i^	0.848 (17)	1.937 (17)	2.6948 (9)	148.0 (16)
N1*A*—H1*A*⋯O1*B* ^ii^	0.880 (15)	1.890 (15)	2.7666 (10)	174.1 (13)
C7*A*—H7*C*⋯O6*B* ^ii^	0.99	2.67	3.4510 (12)	136
C12*A*—H12*D*⋯O2*A* ^iii^	0.98	2.60	3.5237 (12)	157
C13*A*—H13*D*⋯O1*B* ^ii^	0.98	2.59	3.3590 (12)	136
C16*A*—H16*D*⋯O4*A* ^iv^	0.98	2.65	3.3136 (13)	126
C24*A*—H24*E*⋯O4*B* ^v^	0.98	2.43	3.3105 (13)	149
N1*B*—H1⋯O1*A* ^i^	0.888 (15)	2.166 (15)	2.9479 (10)	146.6 (12)
C7*B*—H7*B*⋯O2*A*	0.99	2.69	3.4992 (11)	139
C9*B*—H9*B*⋯O6*A*	0.99	2.59	3.5751 (11)	172
C15*B*—H15*A*⋯O5*B* ^iii^	0.99	2.60	3.4993 (12)	151
C23*B*—H23*B*⋯O2*B* ^vi^	0.98	2.55	3.4277 (13)	149

Symmetry codes: (i) 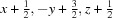; (ii) 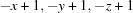; (iii) 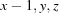; (iv) 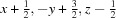; (v) 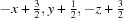; (vi) 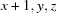.

**Table 3:** Hydrogen-bond geometry (Å, °) for (III)

*D*—H⋯*A*	*D*—H	H⋯*A*	*D*⋯*A*	*D*—H⋯*A*
N1—H1⋯O1^i^	0.90 (2)	1.94 (2)	2.7776 (16)	154.2 (18)
C13—H13*B*⋯O2^ii^	0.98	2.65	3.409 (2)	134
C19—H19⋯N1	0.95	2.48	3.4148 (19)	168

Symmetry codes: (i) 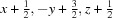; (ii) 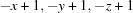.

**Table 4:** Experimental details

	(I)	(II)	(III)
Crystal data			
Chemical formula	C_23_H_27_NO_6_	C_23_H_29_NO_6_	C_29_H_29_NO_3_
*M*_r_	413.45	415.47	439.53
Crystal system, space group	Triclinic, *P* 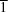	Monoclinic, *P*2_1_/*n*	Monoclinic, *P*2_1_/*n*
Temperature (K)	100	100	100
*a*, *b*, *c* (Å)	8.7024 (3), 9.8709 (4), 13.1621 (5)	10.8854 (4), 25.2446 (10), 15.3665 (6)	11.6527 (3), 18.1986 (4), 12.3435 (3)
α, β, γ (°)	90.3822 (19), 108.9608 (18), 97.3571 (18)	90, 100.7606 (19), 90	90, 114.8758 (12), 90
*V* (Å^3^)	1059.08 (7)	4148.4 (3)	2374.74 (10)
*Z*	2	8	4
Radiation type	Mo *K*α	Mo *K*α	Mo *K*α
μ (mm^−1^)	0.09	0.10	0.08
Crystal size (mm)	0.39 × 0.25 × 0.13	0.48 × 0.43 × 0.31	0.45 × 0.12 × 0.11

Data collection			
Diffractometer	Bruker SMART BREEZE CCD	Bruker SMART BREEZE CCD	Bruker SMART BREEZE CCD
No. of measured, independent and observed [*I* > 2σ(*I*)] reflections	29458, 5315, 4254	168826, 12707, 11044	72579, 5902, 4515
*R*_int_	0.031	0.045	0.055
(sin θ/λ)_max_ (Å^−1^)	0.683	0.716	0.667

Refinement			
*R*[*F* ^2^ > 2σ(*F* ^2^)], *wR*(*F* ^2^), *S*	0.054, 0.154, 1.04	0.039, 0.108, 1.04	0.048, 0.135, 1.04
No. of reflections	5315	12707	5902
No. of parameters	291	569	306
H-atom treatment	H atoms treated by a mixture of independent and constrained refinement	H atoms treated by a mixture of independent and constrained refinement	H atoms treated by a mixture of independent and constrained refinement
Δρ_max_, Δρ_min_ (e Å^−3^)	0.65, −0.25	0.52, −0.21	0.54, −0.22

Computer programs: *APEX2* and *SAINT* (Bruker, 2013 ▸), *SHELXS* (Sheldrick, 2008 ▸), *SHELXL* (Sheldrick, 2015 ▸) and *OLEX2* (Dolomanov *et al.*, 2009 ▸).

## supplementary crystallographic information

### 2,2'-[(2-Nitrophenyl)methylene]bis(3-hydroxy-5,5-dimethylcyclohex-2-enone) (I) Crystal data

**Table:**

C_23_H_27_NO_6_	*Z* = 2
*M**_r_* = 413.45	*F*(000) = 440
Triclinic, *P*1	*D*_x_ = 1.297 Mg m^−^^3^
*a* = 8.7024 (3) Å	Mo *K*α radiation, λ = 0.71073 Å
*b* = 9.8709 (4) Å	Cell parameters from 9400 reflections
*c* = 13.1621 (5) Å	θ = 2.5–28.8°
α = 90.3822 (19)°	µ = 0.09 mm^−^^1^
β = 108.9608 (18)°	*T* = 100 K
γ = 97.3571 (18)°	Prism, yellow
*V* = 1059.08 (7) Å^3^	0.39 × 0.25 × 0.13 mm

### 2,2'-[(2-Nitrophenyl)methylene]bis(3-hydroxy-5,5-dimethylcyclohex-2-enone) (I) Data collection

**Table:**

Bruker SMART BREEZE CCD diffractometer	*R*_int_ = 0.031
φ and ω scans	θ_max_ = 29.1°, θ_min_ = 2.5°
29458 measured reflections	*h* = −11→11
5315 independent reflections	*k* = −13→13
4254 reflections with *I* > 2σ(*I*)	*l* = −17→17

### 2,2'-[(2-Nitrophenyl)methylene]bis(3-hydroxy-5,5-dimethylcyclohex-2-enone) (I) Refinement

**Table:**

Refinement on *F*^2^	Primary atom site location: structure-invariant direct methods
Least-squares matrix: full	Hydrogen site location: mixed
*R*[*F*^2^ > 2σ(*F*^2^)] = 0.054	H atoms treated by a mixture of independent and constrained refinement
*wR*(*F*^2^) = 0.154	*w* = 1/[σ^2^(*F*_o_^2^) + (0.0841*P*)^2^ + 0.5356*P*] where *P* = (*F*_o_^2^ + 2*F*_c_^2^)/3
*S* = 1.04	(Δ/σ)_max_ < 0.001
5315 reflections	Δρ_max_ = 0.65 e Å^−^^3^
291 parameters	Δρ_min_ = −0.25 e Å^−^^3^
0 restraints	

### 2,2'-[(2-Nitrophenyl)methylene]bis(3-hydroxy-5,5-dimethylcyclohex-2-enone) (I) Special details

**Table:**

Geometry. All esds (except the esd in the dihedral angle between two l.s. planes) are estimated using the full covariance matrix. The cell esds are taken into account individually in the estimation of esds in distances, angles and torsion angles; correlations between esds in cell parameters are only used when they are defined by crystal symmetry. An approximate (isotropic) treatment of cell esds is used for estimating esds involving l.s. planes.

### 2,2'-[(2-Nitrophenyl)methylene]bis(3-hydroxy-5,5-dimethylcyclohex-2-enone) (I) Fractional atomic coordinates and isotropic or equivalent isotropic displacement parameters (Å^2^)

**Table:**

	*x*	*y*	*z*	*U*_iso_*/*U*_eq_	
O5	0.04549 (13)	0.40295 (11)	0.15867 (9)	0.0208 (2)	
O3	0.21250 (13)	0.20362 (12)	0.17985 (9)	0.0236 (3)	
O4	0.68127 (15)	0.53646 (13)	0.29954 (11)	0.0320 (3)	
O6	0.50510 (15)	0.73583 (13)	0.29901 (10)	0.0296 (3)	
C9	0.11023 (18)	0.52479 (16)	0.18099 (11)	0.0185 (3)	
O2	0.61739 (15)	0.30380 (15)	0.47643 (10)	0.0346 (3)	
O1	0.50744 (18)	0.09861 (14)	0.41032 (11)	0.0380 (3)	
C16	0.44538 (19)	0.37141 (16)	0.25549 (12)	0.0203 (3)	
C8	0.27365 (18)	0.56290 (16)	0.25447 (11)	0.0183 (3)	
C7	0.37049 (18)	0.45661 (15)	0.31983 (11)	0.0182 (3)	
H7	0.466554	0.510901	0.375098	0.022*	
N1	0.49857 (19)	0.21541 (16)	0.43905 (11)	0.0279 (3)	
C10	0.0114 (2)	0.63357 (17)	0.12509 (13)	0.0235 (3)	
H10A	−0.104225	0.606543	0.120725	0.028*	
H10B	0.014314	0.636767	0.050574	0.028*	
C17	0.36806 (18)	0.25256 (16)	0.19662 (12)	0.0209 (3)	
C13	0.34611 (19)	0.69472 (17)	0.25407 (12)	0.0218 (3)	
C6	0.28101 (18)	0.36902 (16)	0.38462 (11)	0.0191 (3)	
C21	0.61154 (19)	0.42118 (18)	0.26078 (13)	0.0237 (3)	
C18	0.4540 (2)	0.15999 (18)	0.14946 (14)	0.0249 (3)	
C12	0.2536 (2)	0.80723 (17)	0.20066 (13)	0.0249 (3)	
H12A	0.273724	0.823572	0.131642	0.030*	
H12B	0.296854	0.892238	0.246928	0.030*	
C11	0.0687 (2)	0.77713 (16)	0.17870 (13)	0.0230 (3)	
C1	0.3365 (2)	0.25007 (17)	0.43280 (12)	0.0238 (3)	
C2	0.2498 (2)	0.16111 (18)	0.48286 (13)	0.0285 (4)	
H2	0.288304	0.078128	0.509312	0.034*	
C4	0.0550 (2)	0.31725 (19)	0.45528 (13)	0.0277 (4)	
H4	−0.039275	0.344284	0.466757	0.033*	
C19	0.6040 (2)	0.23166 (18)	0.12570 (14)	0.0267 (4)	
C14	0.0311 (2)	0.78794 (18)	0.28423 (14)	0.0280 (4)	
H14A	−0.086246	0.759417	0.270562	0.042*	
H14B	0.060734	0.882821	0.313448	0.042*	
H14C	0.094698	0.728498	0.336144	0.042*	
C5	0.13841 (19)	0.40093 (17)	0.40030 (12)	0.0220 (3)	
H5	0.097129	0.482417	0.372502	0.026*	
C20	0.7057 (2)	0.3260 (2)	0.22307 (15)	0.0310 (4)	
H20A	0.756393	0.268957	0.283196	0.037*	
H20B	0.795669	0.381263	0.205032	0.037*	
C3	0.1069 (2)	0.19581 (19)	0.49334 (14)	0.0304 (4)	
H3A	0.044832	0.136339	0.526547	0.036*	
C22	0.7073 (2)	0.1264 (2)	0.10677 (18)	0.0361 (4)	
H22A	0.740886	0.071941	0.170262	0.054*	
H22B	0.804872	0.173597	0.093904	0.054*	
H22C	0.642313	0.066101	0.043989	0.054*	
C15	−0.0190 (2)	0.87981 (19)	0.10242 (15)	0.0321 (4)	
H15A	0.001219	0.871620	0.033763	0.048*	
H15B	0.022549	0.972726	0.134428	0.048*	
H15C	−0.137191	0.860984	0.090111	0.048*	
C23	0.5504 (3)	0.3151 (2)	0.02618 (16)	0.0377 (4)	
H23A	0.491440	0.253400	−0.036919	0.057*	
H23B	0.647292	0.366841	0.015346	0.057*	
H23C	0.478164	0.378662	0.036190	0.057*	
H3	0.160 (4)	0.282 (3)	0.187 (2)	0.069 (9)*	
H18A	0.377 (3)	0.114 (2)	0.093 (2)	0.042 (6)*	
H18B	0.486 (3)	0.084 (2)	0.2037 (19)	0.042 (6)*	
H6	0.569 (5)	0.647 (4)	0.307 (3)	0.103 (12)*	

### 2,2'-[(2-Nitrophenyl)methylene]bis(3-hydroxy-5,5-dimethylcyclohex-2-enone) (I) Atomic displacement parameters (Å^2^)

**Table:**

	*U*^11^	*U*^22^	*U*^33^	*U*^12^	*U*^13^	*U*^23^
O5	0.0178 (5)	0.0222 (6)	0.0217 (5)	0.0004 (4)	0.0066 (4)	−0.0023 (4)
O3	0.0172 (5)	0.0246 (6)	0.0287 (6)	−0.0011 (4)	0.0089 (4)	−0.0045 (5)
O4	0.0232 (6)	0.0310 (7)	0.0418 (7)	−0.0051 (5)	0.0142 (5)	−0.0105 (5)
O6	0.0217 (6)	0.0284 (6)	0.0351 (7)	−0.0051 (5)	0.0080 (5)	−0.0042 (5)
C9	0.0194 (7)	0.0225 (7)	0.0163 (7)	0.0031 (6)	0.0095 (6)	−0.0003 (5)
O2	0.0232 (6)	0.0471 (8)	0.0292 (6)	0.0049 (6)	0.0029 (5)	0.0016 (6)
O1	0.0496 (8)	0.0338 (7)	0.0369 (7)	0.0206 (6)	0.0170 (6)	0.0081 (6)
C16	0.0182 (7)	0.0224 (7)	0.0216 (7)	0.0027 (6)	0.0083 (6)	−0.0001 (6)
C8	0.0177 (7)	0.0213 (7)	0.0167 (6)	0.0027 (5)	0.0068 (5)	−0.0018 (5)
C7	0.0163 (7)	0.0209 (7)	0.0172 (7)	0.0005 (5)	0.0061 (5)	−0.0027 (5)
N1	0.0304 (8)	0.0335 (8)	0.0209 (7)	0.0114 (6)	0.0070 (6)	0.0047 (6)
C10	0.0233 (8)	0.0249 (8)	0.0202 (7)	0.0034 (6)	0.0043 (6)	0.0017 (6)
C17	0.0178 (7)	0.0246 (8)	0.0211 (7)	0.0024 (6)	0.0075 (6)	0.0004 (6)
C13	0.0210 (7)	0.0249 (8)	0.0203 (7)	−0.0007 (6)	0.0095 (6)	−0.0032 (6)
C6	0.0185 (7)	0.0236 (7)	0.0143 (6)	0.0000 (6)	0.0054 (5)	−0.0029 (5)
C21	0.0181 (7)	0.0310 (9)	0.0213 (7)	0.0006 (6)	0.0068 (6)	−0.0029 (6)
C18	0.0211 (8)	0.0265 (8)	0.0280 (8)	0.0008 (6)	0.0103 (7)	−0.0065 (7)
C12	0.0299 (8)	0.0208 (8)	0.0260 (8)	−0.0003 (6)	0.0135 (7)	0.0017 (6)
C11	0.0278 (8)	0.0218 (8)	0.0210 (7)	0.0052 (6)	0.0095 (6)	0.0034 (6)
C1	0.0234 (8)	0.0280 (8)	0.0186 (7)	0.0048 (6)	0.0044 (6)	−0.0028 (6)
C2	0.0382 (10)	0.0257 (8)	0.0194 (7)	0.0022 (7)	0.0072 (7)	0.0019 (6)
C4	0.0255 (8)	0.0383 (10)	0.0210 (7)	0.0000 (7)	0.0115 (6)	−0.0012 (7)
C19	0.0241 (8)	0.0288 (9)	0.0311 (8)	0.0032 (6)	0.0144 (7)	−0.0028 (7)
C14	0.0333 (9)	0.0289 (9)	0.0278 (8)	0.0079 (7)	0.0166 (7)	0.0018 (7)
C5	0.0224 (8)	0.0264 (8)	0.0179 (7)	0.0037 (6)	0.0076 (6)	0.0005 (6)
C20	0.0176 (7)	0.0369 (10)	0.0395 (10)	−0.0011 (7)	0.0128 (7)	−0.0102 (8)
C3	0.0365 (9)	0.0335 (9)	0.0222 (8)	−0.0030 (7)	0.0142 (7)	0.0013 (7)
C22	0.0283 (9)	0.0356 (10)	0.0493 (11)	0.0062 (7)	0.0190 (8)	−0.0077 (8)
C15	0.0384 (10)	0.0259 (9)	0.0312 (9)	0.0091 (7)	0.0084 (8)	0.0071 (7)
C23	0.0470 (11)	0.0377 (11)	0.0379 (10)	0.0098 (9)	0.0254 (9)	0.0049 (8)

### 2,2'-[(2-Nitrophenyl)methylene]bis(3-hydroxy-5,5-dimethylcyclohex-2-enone) (I) Geometric parameters (Å, º)

**Table:**

O5—C9	1.2507 (19)	C17—C18	1.499 (2)
O3—C17	1.3234 (19)	C13—C12	1.499 (2)
O4—C21	1.240 (2)	C6—C1	1.404 (2)
O6—C13	1.3228 (19)	C6—C5	1.395 (2)
C9—C8	1.437 (2)	C21—C20	1.502 (2)
C9—C10	1.507 (2)	C18—C19	1.528 (2)
O2—N1	1.227 (2)	C12—C11	1.528 (2)
O1—N1	1.231 (2)	C11—C14	1.533 (2)
C16—C7	1.529 (2)	C11—C15	1.527 (2)
C16—C17	1.370 (2)	C1—C2	1.391 (2)
C16—C21	1.445 (2)	C2—C3	1.380 (3)
C8—C7	1.523 (2)	C4—C5	1.389 (2)
C8—C13	1.372 (2)	C4—C3	1.375 (3)
C7—C6	1.533 (2)	C19—C20	1.526 (2)
N1—C1	1.470 (2)	C19—C22	1.526 (2)
C10—C11	1.527 (2)	C19—C23	1.526 (3)
			
O5—C9—C8	122.69 (14)	O4—C21—C16	122.67 (15)
O5—C9—C10	117.37 (13)	O4—C21—C20	118.99 (14)
C8—C9—C10	119.93 (13)	C16—C21—C20	118.26 (15)
C17—C16—C7	124.87 (14)	C17—C18—C19	114.49 (14)
C17—C16—C21	119.05 (14)	C13—C12—C11	113.81 (13)
C21—C16—C7	116.07 (13)	C10—C11—C12	107.37 (13)
C9—C8—C7	121.06 (13)	C10—C11—C14	111.47 (14)
C13—C8—C9	117.49 (14)	C10—C11—C15	109.11 (14)
C13—C8—C7	121.00 (13)	C12—C11—C14	110.01 (14)
C16—C7—C6	112.82 (12)	C15—C11—C12	110.07 (14)
C8—C7—C16	113.54 (12)	C15—C11—C14	108.80 (14)
C8—C7—C6	114.51 (12)	C6—C1—N1	121.11 (15)
O2—N1—O1	124.15 (16)	C2—C1—N1	114.97 (15)
O2—N1—C1	117.60 (14)	C2—C1—C6	123.83 (15)
O1—N1—C1	118.19 (15)	C3—C2—C1	118.67 (16)
C9—C10—C11	115.19 (13)	C3—C4—C5	120.92 (16)
O3—C17—C16	123.87 (14)	C20—C19—C18	107.98 (14)
O3—C17—C18	112.79 (14)	C20—C19—C22	109.38 (15)
C16—C17—C18	123.25 (14)	C22—C19—C18	110.18 (15)
O6—C13—C8	123.82 (15)	C23—C19—C18	110.18 (15)
O6—C13—C12	112.72 (14)	C23—C19—C20	109.85 (16)
C8—C13—C12	123.46 (14)	C23—C19—C22	109.26 (15)
C1—C6—C7	122.42 (13)	C4—C5—C6	122.05 (15)
C5—C6—C7	122.82 (14)	C21—C20—C19	114.88 (14)
C5—C6—C1	114.76 (14)	C4—C3—C2	119.41 (16)
			
O5—C9—C8—C7	−7.3 (2)	C7—C6—C1—C2	−173.36 (14)
O5—C9—C8—C13	165.07 (14)	C7—C6—C5—C4	177.16 (14)
O5—C9—C10—C11	161.40 (13)	N1—C1—C2—C3	171.47 (15)
O3—C17—C18—C19	156.51 (14)	C10—C9—C8—C7	174.32 (13)
O4—C21—C20—C19	−146.42 (16)	C10—C9—C8—C13	−13.3 (2)
O6—C13—C12—C11	−162.31 (13)	C17—C16—C7—C8	−88.49 (18)
C9—C8—C7—C16	78.45 (17)	C17—C16—C7—C6	43.9 (2)
C9—C8—C7—C6	−53.11 (18)	C17—C16—C21—O4	169.75 (16)
C9—C8—C13—O6	−164.77 (14)	C17—C16—C21—C20	−13.6 (2)
C9—C8—C13—C12	14.0 (2)	C17—C18—C19—C20	45.7 (2)
C9—C10—C11—C12	49.40 (17)	C17—C18—C19—C22	165.08 (15)
C9—C10—C11—C14	−71.15 (18)	C17—C18—C19—C23	−74.29 (19)
C9—C10—C11—C15	168.67 (14)	C13—C8—C7—C16	−93.69 (17)
O2—N1—C1—C6	51.4 (2)	C13—C8—C7—C6	134.75 (14)
O2—N1—C1—C2	−125.14 (16)	C13—C12—C11—C10	−48.40 (17)
O1—N1—C1—C6	−131.16 (16)	C13—C12—C11—C14	73.07 (17)
O1—N1—C1—C2	52.2 (2)	C13—C12—C11—C15	−167.05 (14)
C16—C7—C6—C1	35.67 (19)	C6—C1—C2—C3	−5.0 (2)
C16—C7—C6—C5	−144.27 (14)	C21—C16—C7—C8	92.93 (16)
C16—C17—C18—C19	−26.7 (2)	C21—C16—C7—C6	−134.69 (14)
C16—C21—C20—C19	36.8 (2)	C21—C16—C17—O3	−174.70 (14)
C8—C9—C10—C11	−20.2 (2)	C21—C16—C17—C18	8.9 (2)
C8—C7—C6—C1	167.58 (13)	C18—C19—C20—C21	−51.4 (2)
C8—C7—C6—C5	−12.4 (2)	C1—C6—C5—C4	−2.8 (2)
C8—C13—C12—C11	18.8 (2)	C1—C2—C3—C4	−0.7 (2)
C7—C16—C17—O3	6.8 (3)	C5—C6—C1—N1	−169.69 (14)
C7—C16—C17—C18	−169.69 (15)	C5—C6—C1—C2	6.6 (2)
C7—C16—C21—O4	−11.6 (2)	C5—C4—C3—C2	4.3 (3)
C7—C16—C21—C20	165.04 (15)	C3—C4—C5—C6	−2.5 (2)
C7—C8—C13—O6	7.6 (2)	C22—C19—C20—C21	−171.31 (16)
C7—C8—C13—C12	−173.56 (14)	C23—C19—C20—C21	68.8 (2)
C7—C6—C1—N1	10.4 (2)		

### 2,2'-[(2-Nitrophenyl)methylene]bis(3-hydroxy-5,5-dimethylcyclohex-2-enone) (I) Hydrogen-bond geometry (Å, º)

**Table:**

*D*—H···*A*	*D*—H	H···*A*	*D*···*A*	*D*—H···*A*
O3—H3···O5	0.97 (3)	1.62 (3)	2.5570 (16)	162 (3)
O6—H6···O4	1.08 (4)	1.58 (4)	2.6437 (19)	166 (3)
C2—H2···O1^i^	0.95	2.63	3.538 (2)	160
C10—H10*B*···O5^ii^	0.99	2.65	3.6138 (19)	165
C15—H15*B*···O3^iii^	0.98	2.58	3.505 (2)	157
C18—H18*B*···O1	1.04 (2)	2.67 (2)	3.381 (2)	125.5 (16)
C20—H20*B*···O5^iv^	0.99	2.43	3.332 (2)	152

Symmetry codes: (i) −*x*+1, −*y*, −*z*+1; (ii) −*x*, −*y*+1, −*z*; (iii) *x*, *y*+1, *z*; (iv) *x*+1, *y*, *z*.

### Ethyl 4-(4-hydroxy-3,5-dimethoxyphenyl)-2,7,7-trimethyl-5-oxo-1,4,5,6,7,8-hexahydroquinoline-3-carboxylate (II) Crystal data

**Table:**

C_23_H_29_NO_6_	*F*(000) = 1776
*M**_r_* = 415.47	*D*_x_ = 1.330 Mg m^−^^3^
Monoclinic, *P*2_1_/*n*	Mo *K*α radiation, λ = 0.71073 Å
*a* = 10.8854 (4) Å	Cell parameters from 9684 reflections
*b* = 25.2446 (10) Å	θ = 2.7–30.5°
*c* = 15.3665 (6) Å	µ = 0.10 mm^−^^1^
β = 100.7606 (19)°	*T* = 100 K
*V* = 4148.4 (3) Å^3^	Prism, colourless
*Z* = 8	0.48 × 0.43 × 0.31 mm

### Ethyl 4-(4-hydroxy-3,5-dimethoxyphenyl)-2,7,7-trimethyl-5-oxo-1,4,5,6,7,8-hexahydroquinoline-3-carboxylate (II) Data collection

**Table:**

Bruker SMART BREEZE CCD diffractometer	*R*_int_ = 0.045
φ and ω scans	θ_max_ = 30.6°, θ_min_ = 1.6°
168826 measured reflections	*h* = −15→15
12707 independent reflections	*k* = −36→36
11044 reflections with *I* > 2σ(*I*)	*l* = −21→21

### Ethyl 4-(4-hydroxy-3,5-dimethoxyphenyl)-2,7,7-trimethyl-5-oxo-1,4,5,6,7,8-hexahydroquinoline-3-carboxylate (II) Refinement

**Table:**

Refinement on *F*^2^	Primary atom site location: structure-invariant direct methods
Least-squares matrix: full	Hydrogen site location: mixed
*R*[*F*^2^ > 2σ(*F*^2^)] = 0.039	H atoms treated by a mixture of independent and constrained refinement
*wR*(*F*^2^) = 0.108	*w* = 1/[σ^2^(*F*_o_^2^) + (0.060*P*)^2^ + 1.3541*P*] where *P* = (*F*_o_^2^ + 2*F*_c_^2^)/3
*S* = 1.04	(Δ/σ)_max_ = 0.001
12707 reflections	Δρ_max_ = 0.52 e Å^−^^3^
569 parameters	Δρ_min_ = −0.20 e Å^−^^3^
0 restraints	

### Ethyl 4-(4-hydroxy-3,5-dimethoxyphenyl)-2,7,7-trimethyl-5-oxo-1,4,5,6,7,8-hexahydroquinoline-3-carboxylate (II) Special details

**Table:**

Geometry. All esds (except the esd in the dihedral angle between two l.s. planes) are estimated using the full covariance matrix. The cell esds are taken into account individually in the estimation of esds in distances, angles and torsion angles; correlations between esds in cell parameters are only used when they are defined by crystal symmetry. An approximate (isotropic) treatment of cell esds is used for estimating esds involving l.s. planes.

### Ethyl 4-(4-hydroxy-3,5-dimethoxyphenyl)-2,7,7-trimethyl-5-oxo-1,4,5,6,7,8-hexahydroquinoline-3-carboxylate (II) Fractional atomic coordinates and isotropic or equivalent isotropic displacement parameters (Å^2^)

**Table:**

	*x*	*y*	*z*	*U*_iso_*/*U*_eq_	
O1A	0.09761 (6)	0.68726 (3)	0.28219 (5)	0.01536 (13)	
O2A	0.66193 (6)	0.61973 (3)	0.32843 (5)	0.01652 (13)	
O3A	0.54423 (6)	0.69363 (3)	0.31163 (5)	0.01527 (13)	
O4A	0.40914 (7)	0.74895 (3)	0.70657 (5)	0.01676 (14)	
H4C	0.4593 (16)	0.7750 (7)	0.7112 (11)	0.035 (4)*	
O5A	0.31079 (7)	0.65461 (3)	0.68643 (4)	0.01866 (14)	
O6A	0.47618 (6)	0.80349 (3)	0.56333 (5)	0.01668 (14)	
N1A	0.34484 (7)	0.54131 (3)	0.39339 (5)	0.01093 (13)	
H1A	0.3460 (13)	0.5068 (6)	0.4013 (9)	0.021 (3)*	
C2A	0.45480 (8)	0.56515 (3)	0.38033 (5)	0.01037 (15)	
C3A	0.45444 (8)	0.61775 (3)	0.36062 (6)	0.01007 (14)	
C4A	0.33953 (8)	0.65156 (3)	0.36544 (5)	0.00946 (14)	
H4B	0.332567	0.679421	0.318505	0.011*	
C5A	0.22391 (8)	0.61725 (3)	0.34576 (5)	0.00925 (14)	
C6A	0.10568 (8)	0.63990 (3)	0.30421 (5)	0.00999 (14)	
C7A	−0.00768 (8)	0.60412 (3)	0.28469 (6)	0.01162 (15)	
H7C	−0.013501	0.588937	0.224662	0.014*	
H7D	−0.083739	0.625617	0.284713	0.014*	
C8A	−0.00446 (8)	0.55865 (3)	0.35161 (6)	0.01072 (15)	
C9A	0.11952 (8)	0.52866 (3)	0.35798 (6)	0.01071 (15)	
H9C	0.130811	0.504944	0.410176	0.013*	
H9D	0.115282	0.506253	0.304626	0.013*	
C10A	0.23072 (8)	0.56462 (3)	0.36583 (5)	0.00927 (14)	
C11A	−0.01825 (9)	0.58146 (4)	0.44184 (6)	0.01572 (17)	
H11D	0.049914	0.606478	0.462131	0.024*	
H11E	−0.014841	0.552601	0.484888	0.024*	
H11F	−0.098635	0.599854	0.436132	0.024*	
C12A	−0.11296 (9)	0.52050 (4)	0.31995 (7)	0.01625 (17)	
H12D	−0.192403	0.539584	0.314712	0.024*	
H12E	−0.110612	0.491581	0.362771	0.024*	
H12F	−0.105306	0.505920	0.262095	0.024*	
C13A	0.56241 (8)	0.52727 (4)	0.38723 (6)	0.01339 (16)	
H13D	0.543503	0.494967	0.417571	0.020*	
H13E	0.638014	0.543801	0.420913	0.020*	
H13F	0.575997	0.518286	0.327703	0.020*	
C14A	0.56421 (8)	0.64179 (4)	0.33347 (6)	0.01162 (15)	
C15A	0.63909 (9)	0.71842 (4)	0.27011 (6)	0.01674 (17)	
H15C	0.723206	0.707623	0.301464	0.020*	
H15D	0.632745	0.757422	0.274256	0.020*	
C16A	0.62173 (10)	0.70211 (4)	0.17397 (7)	0.02087 (19)	
H16D	0.674331	0.724303	0.143533	0.031*	
H16E	0.533831	0.706570	0.145851	0.031*	
H16F	0.645760	0.664893	0.170167	0.031*	
C17A	0.35248 (8)	0.67935 (3)	0.45549 (6)	0.01023 (15)	
C18A	0.32110 (8)	0.65290 (3)	0.52864 (6)	0.01201 (15)	
H18A	0.286818	0.618226	0.521374	0.014*	
C19A	0.33951 (8)	0.67677 (4)	0.61162 (6)	0.01263 (16)	
C20A	0.39194 (8)	0.72784 (4)	0.62385 (6)	0.01266 (16)	
C21A	0.42098 (8)	0.75402 (3)	0.55069 (6)	0.01235 (16)	
C22A	0.40129 (8)	0.73027 (3)	0.46704 (6)	0.01162 (15)	
H22A	0.421364	0.749009	0.417934	0.014*	
C23A	0.25765 (10)	0.60304 (4)	0.67836 (7)	0.02005 (19)	
H23D	0.179341	0.603652	0.634894	0.030*	
H23E	0.240715	0.591604	0.735908	0.030*	
H23F	0.316250	0.578294	0.658694	0.030*	
C24A	0.38733 (10)	0.84600 (4)	0.54853 (7)	0.02017 (19)	
H24D	0.337479	0.843172	0.488624	0.030*	
H24E	0.431736	0.879953	0.554867	0.030*	
H24F	0.332068	0.843913	0.591977	0.030*	
O1B	0.64618 (7)	0.56795 (3)	0.59302 (5)	0.01898 (14)	
O2B	0.42404 (7)	0.61173 (3)	0.93863 (5)	0.02210 (15)	
O3B	0.47350 (6)	0.54093 (3)	0.86392 (5)	0.01724 (14)	
O4B	1.07150 (7)	0.47496 (3)	0.91945 (5)	0.01745 (14)	
H4	1.1277 (17)	0.4973 (7)	0.9396 (12)	0.040 (5)*	
O5B	1.09254 (6)	0.57723 (3)	0.88846 (5)	0.01832 (14)	
O6B	0.85544 (7)	0.42479 (3)	0.88741 (5)	0.01802 (14)	
N1B	0.67195 (7)	0.70086 (3)	0.81054 (5)	0.01272 (14)	
H1	0.6769 (13)	0.7356 (6)	0.8200 (9)	0.022 (3)*	
C2B	0.60016 (8)	0.67286 (4)	0.86093 (6)	0.01315 (16)	
C3B	0.57467 (8)	0.62086 (4)	0.84315 (6)	0.01213 (15)	
C4B	0.63347 (8)	0.59179 (3)	0.77391 (6)	0.01085 (15)	
H4A	0.570391	0.566145	0.742480	0.013*	
C5B	0.66228 (8)	0.63135 (3)	0.70649 (6)	0.01071 (15)	
C6B	0.66639 (8)	0.61434 (3)	0.61710 (6)	0.01161 (15)	
C7B	0.69106 (8)	0.65543 (3)	0.55138 (6)	0.01271 (15)	
H7A	0.610642	0.671873	0.523744	0.015*	
H7B	0.725884	0.637650	0.503929	0.015*	
C8B	0.78141 (8)	0.69911 (3)	0.59211 (6)	0.01258 (15)	
C9B	0.73133 (8)	0.72350 (3)	0.67009 (6)	0.01263 (15)	
H9A	0.798126	0.745108	0.705925	0.015*	
H9B	0.660884	0.747437	0.646616	0.015*	
C10B	0.68745 (8)	0.68288 (3)	0.72894 (6)	0.01098 (15)	
C11B	0.91219 (9)	0.67533 (4)	0.62358 (7)	0.01783 (18)	
H11A	0.906685	0.645916	0.664297	0.027*	
H11B	0.968228	0.702619	0.654158	0.027*	
H11C	0.945007	0.662303	0.572359	0.027*	
C12B	0.78685 (10)	0.74186 (4)	0.52223 (7)	0.01908 (18)	
H12A	0.817987	0.726305	0.472081	0.029*	
H12B	0.843131	0.770322	0.548282	0.029*	
H12C	0.702898	0.756306	0.501600	0.029*	
C13B	0.55601 (10)	0.70687 (4)	0.92881 (7)	0.02043 (19)	
H13A	0.617141	0.735037	0.947664	0.031*	
H13B	0.546865	0.685161	0.980088	0.031*	
H13C	0.475127	0.722665	0.903090	0.031*	
C14B	0.48511 (8)	0.59282 (4)	0.88766 (6)	0.01479 (16)	
C15B	0.37849 (9)	0.51189 (4)	0.89925 (7)	0.01923 (18)	
H15A	0.294455	0.525795	0.873705	0.023*	
H15B	0.392024	0.516062	0.964409	0.023*	
C16B	0.38731 (10)	0.45419 (4)	0.87558 (7)	0.02153 (19)	
H16A	0.376740	0.450555	0.811117	0.032*	
H16B	0.321567	0.434209	0.896828	0.032*	
H16C	0.469351	0.440325	0.903427	0.032*	
C17B	0.75057 (8)	0.56042 (3)	0.81518 (6)	0.01136 (15)	
C18B	0.86636 (8)	0.58599 (4)	0.83344 (6)	0.01318 (16)	
H18	0.871998	0.622799	0.821919	0.016*	
C19B	0.97326 (8)	0.55741 (4)	0.86850 (6)	0.01334 (16)	
C20B	0.96602 (8)	0.50345 (4)	0.88589 (6)	0.01323 (16)	
C21B	0.85061 (9)	0.47788 (3)	0.86888 (6)	0.01301 (16)	
C22B	0.74257 (8)	0.50638 (4)	0.83344 (6)	0.01278 (16)	
H22	0.663772	0.489024	0.821767	0.015*	
C23B	1.10806 (9)	0.63320 (4)	0.88435 (8)	0.02108 (19)	
H23A	1.077197	0.645485	0.823712	0.032*	
H23B	1.196897	0.642070	0.901795	0.032*	
H23C	1.060743	0.650516	0.924718	0.032*	
C24B	0.74049 (10)	0.39706 (4)	0.88304 (7)	0.01957 (19)	
H24A	0.689946	0.400674	0.823490	0.029*	
H24B	0.694746	0.411935	0.926559	0.029*	
H24C	0.757687	0.359498	0.896071	0.029*	

### Ethyl 4-(4-hydroxy-3,5-dimethoxyphenyl)-2,7,7-trimethyl-5-oxo-1,4,5,6,7,8-hexahydroquinoline-3-carboxylate (II) Atomic displacement parameters (Å^2^)

**Table:**

	*U*^11^	*U*^22^	*U*^33^	*U*^12^	*U*^13^	*U*^23^
O1A	0.0138 (3)	0.0100 (3)	0.0212 (3)	0.0012 (2)	0.0004 (2)	0.0044 (2)
O2A	0.0114 (3)	0.0183 (3)	0.0206 (3)	0.0018 (2)	0.0049 (2)	0.0016 (3)
O3A	0.0153 (3)	0.0116 (3)	0.0209 (3)	−0.0018 (2)	0.0086 (3)	0.0018 (2)
O4A	0.0174 (3)	0.0172 (3)	0.0155 (3)	−0.0066 (3)	0.0026 (2)	−0.0073 (2)
O5A	0.0266 (4)	0.0172 (3)	0.0127 (3)	−0.0096 (3)	0.0049 (3)	−0.0020 (2)
O6A	0.0150 (3)	0.0086 (3)	0.0256 (3)	−0.0037 (2)	0.0018 (3)	−0.0039 (2)
N1A	0.0099 (3)	0.0076 (3)	0.0152 (3)	0.0012 (2)	0.0019 (3)	0.0017 (3)
C2A	0.0098 (3)	0.0115 (4)	0.0096 (3)	0.0008 (3)	0.0012 (3)	−0.0006 (3)
C3A	0.0088 (3)	0.0107 (3)	0.0106 (3)	0.0002 (3)	0.0017 (3)	−0.0005 (3)
C4A	0.0087 (3)	0.0078 (3)	0.0117 (3)	−0.0001 (3)	0.0014 (3)	0.0002 (3)
C5A	0.0088 (3)	0.0084 (3)	0.0104 (3)	0.0001 (3)	0.0016 (3)	0.0004 (3)
C6A	0.0101 (3)	0.0097 (3)	0.0102 (3)	0.0007 (3)	0.0020 (3)	0.0005 (3)
C7A	0.0095 (3)	0.0113 (4)	0.0134 (4)	−0.0005 (3)	0.0004 (3)	0.0006 (3)
C8A	0.0096 (3)	0.0098 (3)	0.0130 (4)	−0.0010 (3)	0.0027 (3)	−0.0007 (3)
C9A	0.0102 (3)	0.0081 (3)	0.0138 (4)	−0.0010 (3)	0.0024 (3)	−0.0005 (3)
C10A	0.0095 (3)	0.0092 (3)	0.0090 (3)	0.0005 (3)	0.0014 (3)	−0.0003 (3)
C11A	0.0166 (4)	0.0174 (4)	0.0146 (4)	−0.0002 (3)	0.0064 (3)	−0.0019 (3)
C12A	0.0119 (4)	0.0147 (4)	0.0220 (4)	−0.0042 (3)	0.0026 (3)	−0.0009 (3)
C13A	0.0118 (4)	0.0126 (4)	0.0159 (4)	0.0035 (3)	0.0031 (3)	0.0003 (3)
C14A	0.0113 (4)	0.0126 (4)	0.0107 (3)	−0.0014 (3)	0.0015 (3)	−0.0008 (3)
C15A	0.0160 (4)	0.0162 (4)	0.0195 (4)	−0.0051 (3)	0.0072 (3)	0.0022 (3)
C16A	0.0203 (5)	0.0245 (5)	0.0190 (4)	−0.0016 (4)	0.0066 (4)	0.0024 (4)
C17A	0.0086 (3)	0.0089 (3)	0.0130 (4)	0.0004 (3)	0.0014 (3)	−0.0009 (3)
C18A	0.0124 (4)	0.0098 (4)	0.0136 (4)	−0.0019 (3)	0.0020 (3)	−0.0013 (3)
C19A	0.0124 (4)	0.0120 (4)	0.0135 (4)	−0.0022 (3)	0.0024 (3)	−0.0008 (3)
C20A	0.0104 (4)	0.0123 (4)	0.0149 (4)	−0.0013 (3)	0.0015 (3)	−0.0040 (3)
C21A	0.0100 (4)	0.0082 (3)	0.0185 (4)	−0.0016 (3)	0.0017 (3)	−0.0027 (3)
C22A	0.0107 (3)	0.0091 (3)	0.0150 (4)	−0.0004 (3)	0.0023 (3)	−0.0003 (3)
C23A	0.0280 (5)	0.0149 (4)	0.0177 (4)	−0.0065 (4)	0.0052 (4)	0.0013 (3)
C24A	0.0219 (5)	0.0102 (4)	0.0281 (5)	0.0000 (3)	0.0039 (4)	−0.0003 (3)
O1B	0.0299 (4)	0.0093 (3)	0.0169 (3)	−0.0001 (3)	0.0023 (3)	−0.0026 (2)
O2B	0.0220 (4)	0.0223 (4)	0.0258 (4)	0.0014 (3)	0.0142 (3)	0.0004 (3)
O3B	0.0148 (3)	0.0159 (3)	0.0229 (3)	−0.0016 (2)	0.0082 (3)	0.0013 (3)
O4B	0.0143 (3)	0.0165 (3)	0.0203 (3)	0.0059 (3)	0.0001 (3)	0.0028 (3)
O5B	0.0112 (3)	0.0149 (3)	0.0272 (4)	0.0005 (2)	−0.0007 (3)	−0.0011 (3)
O6B	0.0181 (3)	0.0111 (3)	0.0256 (4)	0.0029 (2)	0.0060 (3)	0.0060 (3)
N1B	0.0151 (3)	0.0098 (3)	0.0137 (3)	0.0001 (3)	0.0038 (3)	−0.0031 (3)
C2B	0.0121 (4)	0.0147 (4)	0.0128 (4)	0.0025 (3)	0.0027 (3)	−0.0008 (3)
C3B	0.0101 (4)	0.0136 (4)	0.0129 (4)	0.0022 (3)	0.0026 (3)	0.0010 (3)
C4B	0.0107 (4)	0.0095 (3)	0.0123 (4)	0.0011 (3)	0.0017 (3)	0.0001 (3)
C5B	0.0105 (3)	0.0094 (3)	0.0119 (4)	0.0012 (3)	0.0014 (3)	−0.0003 (3)
C6B	0.0113 (4)	0.0099 (4)	0.0128 (4)	0.0016 (3)	0.0001 (3)	−0.0009 (3)
C7B	0.0147 (4)	0.0114 (4)	0.0117 (4)	−0.0004 (3)	0.0017 (3)	−0.0008 (3)
C8B	0.0127 (4)	0.0122 (4)	0.0131 (4)	−0.0004 (3)	0.0032 (3)	−0.0011 (3)
C9B	0.0143 (4)	0.0093 (4)	0.0147 (4)	−0.0006 (3)	0.0037 (3)	−0.0013 (3)
C10B	0.0098 (3)	0.0107 (4)	0.0122 (4)	0.0015 (3)	0.0014 (3)	−0.0011 (3)
C11B	0.0133 (4)	0.0205 (4)	0.0197 (4)	0.0007 (3)	0.0032 (3)	−0.0036 (3)
C12B	0.0230 (5)	0.0174 (4)	0.0177 (4)	−0.0045 (4)	0.0061 (4)	0.0020 (3)
C13B	0.0250 (5)	0.0196 (5)	0.0191 (4)	0.0027 (4)	0.0105 (4)	−0.0044 (4)
C14B	0.0122 (4)	0.0162 (4)	0.0162 (4)	0.0020 (3)	0.0030 (3)	0.0030 (3)
C15B	0.0148 (4)	0.0195 (4)	0.0252 (5)	−0.0026 (3)	0.0082 (4)	0.0042 (4)
C16B	0.0210 (5)	0.0193 (5)	0.0244 (5)	−0.0038 (4)	0.0046 (4)	0.0020 (4)
C17B	0.0117 (4)	0.0108 (4)	0.0116 (4)	0.0020 (3)	0.0023 (3)	−0.0005 (3)
C18B	0.0131 (4)	0.0106 (4)	0.0152 (4)	0.0014 (3)	0.0012 (3)	−0.0006 (3)
C19B	0.0118 (4)	0.0138 (4)	0.0140 (4)	0.0009 (3)	0.0012 (3)	−0.0016 (3)
C20B	0.0138 (4)	0.0139 (4)	0.0119 (4)	0.0046 (3)	0.0021 (3)	0.0008 (3)
C21B	0.0166 (4)	0.0107 (4)	0.0126 (4)	0.0028 (3)	0.0049 (3)	0.0016 (3)
C22B	0.0135 (4)	0.0120 (4)	0.0134 (4)	0.0015 (3)	0.0041 (3)	0.0004 (3)
C23B	0.0156 (4)	0.0162 (4)	0.0307 (5)	−0.0016 (3)	0.0025 (4)	−0.0033 (4)
C24B	0.0213 (5)	0.0145 (4)	0.0243 (5)	−0.0009 (3)	0.0078 (4)	0.0041 (3)

### Ethyl 4-(4-hydroxy-3,5-dimethoxyphenyl)-2,7,7-trimethyl-5-oxo-1,4,5,6,7,8-hexahydroquinoline-3-carboxylate (II) Geometric parameters (Å, º)

**Table:**

O1A—C6A	1.2412 (10)	O1B—C6B	1.2355 (11)
O2A—C14A	1.2159 (11)	O2B—C14B	1.2156 (12)
O3A—C14A	1.3583 (11)	O3B—C14B	1.3593 (12)
O3A—C15A	1.4529 (11)	O3B—C15B	1.4530 (11)
O4A—C20A	1.3587 (11)	O4B—C20B	1.3713 (11)
O5A—C19A	1.3661 (11)	O5B—C19B	1.3717 (11)
O5A—C23A	1.4206 (12)	O5B—C23B	1.4257 (12)
O6A—C21A	1.3837 (10)	O6B—C21B	1.3690 (11)
O6A—C24A	1.4341 (12)	O6B—C24B	1.4245 (12)
N1A—C2A	1.3874 (11)	N1B—C2B	1.3913 (12)
N1A—C10A	1.3683 (11)	N1B—C10B	1.3733 (11)
C2A—C3A	1.3617 (12)	C2B—C3B	1.3589 (13)
C2A—C13A	1.5006 (12)	C2B—C13B	1.4979 (13)
C3A—C4A	1.5275 (12)	C3B—C4B	1.5280 (12)
C3A—C14A	1.4684 (12)	C3B—C14B	1.4725 (13)
C4A—C5A	1.5112 (11)	C4B—C5B	1.5134 (12)
C4A—C17A	1.5344 (12)	C4B—C17B	1.5337 (12)
C5A—C6A	1.4437 (11)	C5B—C6B	1.4479 (12)
C5A—C10A	1.3628 (11)	C5B—C10B	1.3604 (12)
C6A—C7A	1.5134 (12)	C6B—C7B	1.5062 (12)
C7A—C8A	1.5371 (12)	C7B—C8B	1.5314 (12)
C8A—C9A	1.5346 (12)	C8B—C9B	1.5350 (12)
C8A—C11A	1.5342 (12)	C8B—C11B	1.5374 (13)
C8A—C12A	1.5313 (12)	C8B—C12B	1.5313 (13)
C9A—C10A	1.4998 (11)	C9B—C10B	1.5027 (12)
C15A—C16A	1.5111 (14)	C15B—C16B	1.5088 (15)
C17A—C18A	1.4034 (12)	C17B—C18B	1.3972 (12)
C17A—C22A	1.3895 (11)	C17B—C22B	1.3987 (12)
C18A—C19A	1.3906 (12)	C18B—C19B	1.3896 (12)
C19A—C20A	1.4082 (12)	C19B—C20B	1.3932 (13)
C20A—C21A	1.3904 (13)	C20B—C21B	1.3931 (13)
C21A—C22A	1.3981 (12)	C21B—C22B	1.3994 (12)
			
C14A—O3A—C15A	115.43 (7)	C14B—O3B—C15B	115.02 (7)
C19A—O5A—C23A	117.08 (7)	C19B—O5B—C23B	117.82 (7)
C21A—O6A—C24A	113.00 (7)	C21B—O6B—C24B	118.16 (7)
C10A—N1A—C2A	121.78 (7)	C10B—N1B—C2B	121.19 (8)
N1A—C2A—C13A	113.36 (7)	N1B—C2B—C13B	112.51 (8)
C3A—C2A—N1A	119.23 (8)	C3B—C2B—N1B	119.59 (8)
C3A—C2A—C13A	127.37 (8)	C3B—C2B—C13B	127.86 (9)
C2A—C3A—C4A	120.17 (7)	C2B—C3B—C4B	120.50 (8)
C2A—C3A—C14A	119.84 (8)	C2B—C3B—C14B	119.94 (8)
C14A—C3A—C4A	119.99 (7)	C14B—C3B—C4B	119.50 (8)
C3A—C4A—C17A	111.17 (7)	C3B—C4B—C17B	112.57 (7)
C5A—C4A—C3A	109.20 (7)	C5B—C4B—C3B	109.09 (7)
C5A—C4A—C17A	112.05 (7)	C5B—C4B—C17B	111.19 (7)
C6A—C5A—C4A	120.12 (7)	C6B—C5B—C4B	120.05 (7)
C10A—C5A—C4A	120.36 (7)	C10B—C5B—C4B	121.01 (8)
C10A—C5A—C6A	119.51 (7)	C10B—C5B—C6B	118.93 (8)
O1A—C6A—C5A	120.93 (8)	O1B—C6B—C5B	122.00 (8)
O1A—C6A—C7A	120.49 (8)	O1B—C6B—C7B	119.74 (8)
C5A—C6A—C7A	118.54 (7)	C5B—C6B—C7B	118.21 (7)
C6A—C7A—C8A	113.41 (7)	C6B—C7B—C8B	113.78 (7)
C9A—C8A—C7A	108.72 (7)	C7B—C8B—C9B	108.25 (7)
C11A—C8A—C7A	109.30 (7)	C7B—C8B—C11B	109.50 (7)
C11A—C8A—C9A	111.15 (7)	C9B—C8B—C11B	110.73 (7)
C12A—C8A—C7A	109.71 (7)	C12B—C8B—C7B	109.08 (7)
C12A—C8A—C9A	109.08 (7)	C12B—C8B—C9B	109.31 (7)
C12A—C8A—C11A	108.87 (7)	C12B—C8B—C11B	109.94 (8)
C10A—C9A—C8A	113.17 (7)	C10B—C9B—C8B	113.28 (7)
N1A—C10A—C9A	115.91 (7)	N1B—C10B—C9B	115.54 (7)
C5A—C10A—N1A	119.81 (8)	C5B—C10B—N1B	119.61 (8)
C5A—C10A—C9A	124.26 (7)	C5B—C10B—C9B	124.85 (8)
O2A—C14A—O3A	121.80 (8)	O2B—C14B—O3B	121.11 (9)
O2A—C14A—C3A	126.64 (8)	O2B—C14B—C3B	126.85 (9)
O3A—C14A—C3A	111.55 (7)	O3B—C14B—C3B	112.03 (8)
O3A—C15A—C16A	110.04 (8)	O3B—C15B—C16B	108.38 (8)
C18A—C17A—C4A	120.65 (7)	C18B—C17B—C4B	119.57 (8)
C22A—C17A—C4A	120.45 (8)	C18B—C17B—C22B	119.86 (8)
C22A—C17A—C18A	118.83 (8)	C22B—C17B—C4B	120.56 (8)
C19A—C18A—C17A	120.88 (8)	C19B—C18B—C17B	119.78 (8)
O5A—C19A—C18A	125.29 (8)	O5B—C19B—C18B	125.90 (8)
O5A—C19A—C20A	114.41 (8)	O5B—C19B—C20B	113.51 (8)
C18A—C19A—C20A	120.30 (8)	C18B—C19B—C20B	120.59 (8)
O4A—C20A—C19A	117.75 (8)	O4B—C20B—C19B	120.77 (8)
O4A—C20A—C21A	123.89 (8)	O4B—C20B—C21B	119.34 (8)
C21A—C20A—C19A	118.35 (8)	C21B—C20B—C19B	119.89 (8)
O6A—C21A—C20A	118.27 (8)	O6B—C21B—C20B	114.60 (8)
O6A—C21A—C22A	120.27 (8)	O6B—C21B—C22B	125.54 (8)
C20A—C21A—C22A	121.37 (8)	C20B—C21B—C22B	119.85 (8)
C17A—C22A—C21A	120.23 (8)	C17B—C22B—C21B	120.02 (8)
			
O1A—C6A—C7A—C8A	−151.01 (8)	O1B—C6B—C7B—C8B	−147.46 (8)
O4A—C20A—C21A—O6A	3.22 (13)	O4B—C20B—C21B—O6B	−0.68 (12)
O4A—C20A—C21A—C22A	179.97 (8)	O4B—C20B—C21B—C22B	−179.08 (8)
O5A—C19A—C20A—O4A	0.60 (12)	O5B—C19B—C20B—O4B	−0.23 (12)
O5A—C19A—C20A—C21A	−178.29 (8)	O5B—C19B—C20B—C21B	179.96 (8)
O6A—C21A—C22A—C17A	176.30 (8)	O6B—C21B—C22B—C17B	−178.23 (8)
N1A—C2A—C3A—C4A	8.37 (12)	N1B—C2B—C3B—C4B	5.35 (13)
N1A—C2A—C3A—C14A	−170.77 (8)	N1B—C2B—C3B—C14B	−171.69 (8)
C2A—N1A—C10A—C5A	−16.97 (12)	C2B—N1B—C10B—C5B	−17.81 (13)
C2A—N1A—C10A—C9A	161.69 (8)	C2B—N1B—C10B—C9B	161.71 (8)
C2A—C3A—C4A—C5A	−29.30 (10)	C2B—C3B—C4B—C5B	−26.83 (11)
C2A—C3A—C4A—C17A	94.83 (9)	C2B—C3B—C4B—C17B	97.09 (10)
C2A—C3A—C14A—O2A	−2.27 (14)	C2B—C3B—C14B—O2B	3.16 (15)
C2A—C3A—C14A—O3A	176.45 (8)	C2B—C3B—C14B—O3B	−178.35 (8)
C3A—C4A—C5A—C6A	−149.71 (8)	C3B—C4B—C5B—C6B	−153.27 (8)
C3A—C4A—C5A—C10A	29.31 (10)	C3B—C4B—C5B—C10B	28.25 (11)
C3A—C4A—C17A—C18A	−83.76 (9)	C3B—C4B—C17B—C18B	−84.89 (10)
C3A—C4A—C17A—C22A	93.08 (9)	C3B—C4B—C17B—C22B	96.04 (9)
C4A—C3A—C14A—O2A	178.59 (8)	C4B—C3B—C14B—O2B	−173.92 (9)
C4A—C3A—C14A—O3A	−2.69 (11)	C4B—C3B—C14B—O3B	4.58 (11)
C4A—C5A—C6A—O1A	1.72 (12)	C4B—C5B—C6B—O1B	0.45 (13)
C4A—C5A—C6A—C7A	179.35 (7)	C4B—C5B—C6B—C7B	177.94 (7)
C4A—C5A—C10A—N1A	−8.32 (12)	C4B—C5B—C10B—N1B	−7.91 (12)
C4A—C5A—C10A—C9A	173.14 (7)	C4B—C5B—C10B—C9B	172.62 (8)
C4A—C17A—C18A—C19A	176.22 (8)	C4B—C17B—C18B—C19B	−178.18 (8)
C4A—C17A—C22A—C21A	−175.56 (8)	C4B—C17B—C22B—C21B	178.27 (8)
C5A—C4A—C17A—C18A	38.74 (10)	C5B—C4B—C17B—C18B	37.86 (11)
C5A—C4A—C17A—C22A	−144.43 (8)	C5B—C4B—C17B—C22B	−141.21 (8)
C5A—C6A—C7A—C8A	31.35 (11)	C5B—C6B—C7B—C8B	34.99 (11)
C6A—C5A—C10A—N1A	170.71 (8)	C6B—C5B—C10B—N1B	173.59 (8)
C6A—C5A—C10A—C9A	−7.84 (12)	C6B—C5B—C10B—C9B	−5.88 (13)
C6A—C7A—C8A—C9A	−53.22 (9)	C6B—C7B—C8B—C9B	−54.30 (10)
C6A—C7A—C8A—C11A	68.27 (9)	C6B—C7B—C8B—C11B	66.51 (10)
C6A—C7A—C8A—C12A	−172.43 (7)	C6B—C7B—C8B—C12B	−173.14 (8)
C7A—C8A—C9A—C10A	45.78 (9)	C7B—C8B—C9B—C10B	44.60 (10)
C8A—C9A—C10A—N1A	164.48 (7)	C8B—C9B—C10B—N1B	164.10 (8)
C8A—C9A—C10A—C5A	−16.93 (12)	C8B—C9B—C10B—C5B	−16.41 (12)
C10A—N1A—C2A—C3A	16.82 (12)	C10B—N1B—C2B—C3B	19.06 (13)
C10A—N1A—C2A—C13A	−161.19 (8)	C10B—N1B—C2B—C13B	−158.87 (8)
C10A—C5A—C6A—O1A	−177.31 (8)	C10B—C5B—C6B—O1B	178.96 (9)
C10A—C5A—C6A—C7A	0.32 (12)	C10B—C5B—C6B—C7B	−3.55 (12)
C11A—C8A—C9A—C10A	−74.57 (9)	C11B—C8B—C9B—C10B	−75.44 (9)
C12A—C8A—C9A—C10A	165.38 (7)	C12B—C8B—C9B—C10B	163.30 (8)
C13A—C2A—C3A—C4A	−173.92 (8)	C13B—C2B—C3B—C4B	−177.07 (9)
C13A—C2A—C3A—C14A	6.94 (13)	C13B—C2B—C3B—C14B	5.89 (14)
C14A—O3A—C15A—C16A	77.56 (10)	C14B—O3B—C15B—C16B	−173.74 (8)
C14A—C3A—C4A—C5A	149.84 (7)	C14B—C3B—C4B—C5B	150.23 (8)
C14A—C3A—C4A—C17A	−86.04 (9)	C14B—C3B—C4B—C17B	−85.86 (10)
C15A—O3A—C14A—O2A	8.56 (12)	C15B—O3B—C14B—O2B	3.78 (13)
C15A—O3A—C14A—C3A	−170.22 (7)	C15B—O3B—C14B—C3B	−174.82 (8)
C17A—C4A—C5A—C6A	86.68 (9)	C17B—C4B—C5B—C6B	82.01 (10)
C17A—C4A—C5A—C10A	−94.30 (9)	C17B—C4B—C5B—C10B	−96.48 (9)
C17A—C18A—C19A—O5A	179.24 (8)	C17B—C18B—C19B—O5B	179.14 (8)
C17A—C18A—C19A—C20A	−0.95 (13)	C17B—C18B—C19B—C20B	−0.19 (13)
C18A—C17A—C22A—C21A	1.33 (13)	C18B—C17B—C22B—C21B	−0.80 (13)
C18A—C19A—C20A—O4A	−179.24 (8)	C18B—C19B—C20B—O4B	179.18 (8)
C18A—C19A—C20A—C21A	1.87 (13)	C18B—C19B—C20B—C21B	−0.63 (13)
C19A—C20A—C21A—O6A	−177.97 (8)	C19B—C20B—C21B—O6B	179.13 (8)
C19A—C20A—C21A—C22A	−1.22 (13)	C19B—C20B—C21B—C22B	0.72 (13)
C20A—C21A—C22A—C17A	−0.39 (13)	C20B—C21B—C22B—C17B	−0.02 (13)
C22A—C17A—C18A—C19A	−0.67 (13)	C22B—C17B—C18B—C19B	0.89 (13)
C23A—O5A—C19A—C18A	−0.11 (14)	C23B—O5B—C19B—C18B	8.86 (14)
C23A—O5A—C19A—C20A	−179.94 (8)	C23B—O5B—C19B—C20B	−171.77 (9)
C24A—O6A—C21A—C20A	−93.57 (10)	C24B—O6B—C21B—C20B	171.90 (8)
C24A—O6A—C21A—C22A	89.64 (10)	C24B—O6B—C21B—C22B	−9.80 (13)

### Ethyl 4-(4-hydroxy-3,5-dimethoxyphenyl)-2,7,7-trimethyl-5-oxo-1,4,5,6,7,8-hexahydroquinoline-3-carboxylate (II) Hydrogen-bond geometry (Å, º)

**Table:**

*D*—H···*A*	*D*—H	H···*A*	*D*···*A*	*D*—H···*A*
O4*A*—H4*C*···O1*A*^i^	0.848 (17)	1.937 (17)	2.6948 (9)	148.0 (16)
N1*A*—H1*A*···O1*B*^ii^	0.880 (15)	1.890 (15)	2.7666 (10)	174.1 (13)
C7*A*—H7*C*···O6*B*^ii^	0.99	2.67	3.4510 (12)	136
C12*A*—H12*D*···O2*A*^iii^	0.98	2.60	3.5237 (12)	157
C13*A*—H13*D*···O1*B*^ii^	0.98	2.59	3.3590 (12)	136
C16*A*—H16*D*···O4*A*^iv^	0.98	2.65	3.3136 (13)	126
C24*A*—H24*E*···O4*B*^v^	0.98	2.43	3.3105 (13)	149
N1*B*—H1···O1*A*^i^	0.888 (15)	2.166 (15)	2.9479 (10)	146.6 (12)
C7*B*—H7*B*···O2*A*	0.99	2.69	3.4992 (11)	139
C9*B*—H9*B*···O6*A*	0.99	2.59	3.5751 (11)	172
C15*B*—H15*A*···O5*B*^iii^	0.99	2.60	3.4993 (12)	151
C23*B*—H23*B*···O2*B*^vi^	0.98	2.55	3.4277 (13)	149

Symmetry codes: (i) *x*+1/2, −*y*+3/2, *z*+1/2; (ii) −*x*+1, −*y*+1, −*z*+1; (iii) *x*−1, *y*, *z*; (iv) *x*+1/2, −*y*+3/2, *z*−1/2; (v) −*x*+3/2, *y*+1/2, −*z*+3/2; (vi) *x*+1, *y*, *z*.

### Ethyl 4-(anthracen-9-yl)-2,7,7-trimethyl-5-oxo-1,4,5,6,7,8-hexahydroquinoline-3-carboxylate (III) Crystal data

**Table:**

C_29_H_29_NO_3_	*F*(000) = 936
*M**_r_* = 439.53	*D*_x_ = 1.229 Mg m^−^^3^
Monoclinic, *P*2_1_/*n*	Mo *K*α radiation, λ = 0.71073 Å
*a* = 11.6527 (3) Å	Cell parameters from 9872 reflections
*b* = 18.1986 (4) Å	θ = 2.2–27.6°
*c* = 12.3435 (3) Å	µ = 0.08 mm^−^^1^
β = 114.8758 (12)°	*T* = 100 K
*V* = 2374.74 (10) Å^3^	Prism, yellow
*Z* = 4	0.45 × 0.12 × 0.11 mm

### Ethyl 4-(anthracen-9-yl)-2,7,7-trimethyl-5-oxo-1,4,5,6,7,8-hexahydroquinoline-3-carboxylate (III) Data collection

**Table:**

Bruker SMART BREEZE CCD diffractometer	*R*_int_ = 0.055
φ and ω scans	θ_max_ = 28.3°, θ_min_ = 2.1°
72579 measured reflections	*h* = −15→15
5902 independent reflections	*k* = −24→24
4515 reflections with *I* > 2σ(*I*)	*l* = −16→16

### Ethyl 4-(anthracen-9-yl)-2,7,7-trimethyl-5-oxo-1,4,5,6,7,8-hexahydroquinoline-3-carboxylate (III) Refinement

**Table:**

Refinement on *F*^2^	Primary atom site location: structure-invariant direct methods
Least-squares matrix: full	Hydrogen site location: mixed
*R*[*F*^2^ > 2σ(*F*^2^)] = 0.048	H atoms treated by a mixture of independent and constrained refinement
*wR*(*F*^2^) = 0.135	*w* = 1/[σ^2^(*F*_o_^2^) + (0.067*P*)^2^ + 1.0544*P*] where *P* = (*F*_o_^2^ + 2*F*_c_^2^)/3
*S* = 1.04	(Δ/σ)_max_ < 0.001
5902 reflections	Δρ_max_ = 0.54 e Å^−^^3^
306 parameters	Δρ_min_ = −0.22 e Å^−^^3^
0 restraints	

### Ethyl 4-(anthracen-9-yl)-2,7,7-trimethyl-5-oxo-1,4,5,6,7,8-hexahydroquinoline-3-carboxylate (III) Special details

**Table:**

Geometry. All esds (except the esd in the dihedral angle between two l.s. planes) are estimated using the full covariance matrix. The cell esds are taken into account individually in the estimation of esds in distances, angles and torsion angles; correlations between esds in cell parameters are only used when they are defined by crystal symmetry. An approximate (isotropic) treatment of cell esds is used for estimating esds involving l.s. planes.

### Ethyl 4-(anthracen-9-yl)-2,7,7-trimethyl-5-oxo-1,4,5,6,7,8-hexahydroquinoline-3-carboxylate (III) Fractional atomic coordinates and isotropic or equivalent isotropic displacement parameters (Å^2^)

**Table:**

	*x*	*y*	*z*	*U*_iso_*/*U*_eq_	
O1	0.34009 (10)	0.80683 (6)	0.16928 (9)	0.0235 (2)	
O2	0.31457 (11)	0.54998 (7)	0.51900 (11)	0.0331 (3)	
O3	0.19449 (10)	0.64051 (6)	0.40339 (10)	0.0256 (2)	
N1	0.61943 (12)	0.65569 (7)	0.47273 (11)	0.0182 (3)	
H1	0.6990 (19)	0.6551 (11)	0.5295 (18)	0.032 (5)*	
C2	0.52536 (13)	0.62310 (7)	0.49757 (12)	0.0177 (3)	
C3	0.40249 (13)	0.63374 (7)	0.42341 (12)	0.0169 (3)	
C4	0.36056 (13)	0.68248 (7)	0.31263 (12)	0.0153 (3)	
H4	0.304711	0.721153	0.322453	0.018*	
C5	0.47314 (13)	0.72274 (7)	0.30848 (12)	0.0152 (3)	
C6	0.44911 (13)	0.78495 (7)	0.22944 (12)	0.0170 (3)	
C7	0.55990 (14)	0.82620 (8)	0.22492 (13)	0.0202 (3)	
H7A	0.575271	0.870831	0.274940	0.024*	
H7B	0.536301	0.842425	0.141710	0.024*	
C8	0.68311 (14)	0.78268 (8)	0.26676 (13)	0.0199 (3)	
C9	0.70717 (13)	0.74830 (8)	0.38790 (13)	0.0188 (3)	
H9A	0.778956	0.713567	0.410903	0.023*	
H9B	0.731471	0.787529	0.449080	0.023*	
C10	0.59379 (13)	0.70822 (7)	0.38663 (12)	0.0158 (3)	
C11	0.67348 (15)	0.72310 (9)	0.17535 (14)	0.0260 (3)	
H11A	0.602860	0.690143	0.164169	0.039*	
H11B	0.752376	0.694870	0.204489	0.039*	
H11C	0.659039	0.746243	0.098998	0.039*	
C12	0.79290 (15)	0.83464 (9)	0.28370 (15)	0.0262 (3)	
H12A	0.872397	0.806942	0.314765	0.039*	
H12B	0.797430	0.873461	0.340391	0.039*	
H12C	0.779071	0.856745	0.206789	0.039*	
C13	0.57721 (15)	0.58079 (8)	0.61266 (13)	0.0240 (3)	
H13A	0.663101	0.597854	0.662873	0.036*	
H13B	0.579314	0.528329	0.595650	0.036*	
H13C	0.523073	0.588557	0.654582	0.036*	
C14	0.30380 (14)	0.60270 (8)	0.45520 (13)	0.0215 (3)	
C15	0.08506 (16)	0.61002 (11)	0.41441 (16)	0.0340 (4)	
H15A	0.023216	0.649703	0.404172	0.041*	
H15B	0.111372	0.589143	0.495409	0.041*	
C16	0.0228 (2)	0.55080 (12)	0.32261 (18)	0.0466 (5)	
H16A	0.080715	0.509048	0.338088	0.070*	
H16B	0.002429	0.570354	0.242627	0.070*	
H16C	−0.055015	0.534573	0.327545	0.070*	
C17	0.27883 (12)	0.64059 (7)	0.19723 (12)	0.0149 (3)	
C18	0.33088 (13)	0.58220 (7)	0.15614 (12)	0.0151 (3)	
C19	0.46066 (13)	0.55974 (8)	0.21467 (12)	0.0171 (3)	
H19	0.515995	0.585501	0.283915	0.021*	
C20	0.50692 (14)	0.50254 (8)	0.17387 (13)	0.0199 (3)	
H20	0.593432	0.489206	0.215031	0.024*	
C21	0.42774 (15)	0.46271 (8)	0.07091 (14)	0.0223 (3)	
H21	0.460922	0.422798	0.043432	0.027*	
C22	0.30435 (14)	0.48181 (8)	0.01176 (13)	0.0206 (3)	
H22	0.251852	0.455228	−0.057734	0.025*	
C23	0.25166 (13)	0.54115 (8)	0.05188 (12)	0.0174 (3)	
C24	0.12363 (14)	0.55804 (8)	−0.00657 (12)	0.0192 (3)	
H24	0.071627	0.530269	−0.074803	0.023*	
C25	0.06980 (13)	0.61454 (8)	0.03234 (12)	0.0178 (3)	
C26	0.14856 (13)	0.65780 (7)	0.13450 (12)	0.0162 (3)	
C27	0.08637 (13)	0.71596 (8)	0.16756 (13)	0.0191 (3)	
H27	0.135306	0.747691	0.231472	0.023*	
C28	−0.04097 (14)	0.72696 (8)	0.10984 (14)	0.0220 (3)	
H28	−0.078874	0.765462	0.135365	0.026*	
C29	−0.11792 (14)	0.68199 (8)	0.01238 (14)	0.0228 (3)	
H29	−0.206831	0.689377	−0.025495	0.027*	
C30	−0.06317 (14)	0.62827 (8)	−0.02621 (13)	0.0210 (3)	
H30	−0.114157	0.599310	−0.093293	0.025*	

### Ethyl 4-(anthracen-9-yl)-2,7,7-trimethyl-5-oxo-1,4,5,6,7,8-hexahydroquinoline-3-carboxylate (III) Atomic displacement parameters (Å^2^)

**Table:**

	*U*^11^	*U*^22^	*U*^33^	*U*^12^	*U*^13^	*U*^23^
O1	0.0196 (5)	0.0252 (5)	0.0203 (5)	0.0012 (4)	0.0030 (4)	0.0046 (4)
O2	0.0326 (7)	0.0324 (6)	0.0380 (7)	−0.0054 (5)	0.0184 (6)	0.0102 (5)
O3	0.0203 (5)	0.0324 (6)	0.0278 (6)	−0.0043 (4)	0.0139 (5)	0.0013 (5)
N1	0.0150 (6)	0.0202 (6)	0.0164 (6)	−0.0014 (5)	0.0038 (5)	0.0016 (5)
C2	0.0210 (7)	0.0160 (6)	0.0158 (6)	−0.0025 (5)	0.0074 (6)	−0.0012 (5)
C3	0.0198 (7)	0.0171 (6)	0.0147 (6)	−0.0025 (5)	0.0082 (5)	−0.0011 (5)
C4	0.0146 (6)	0.0163 (6)	0.0147 (6)	−0.0009 (5)	0.0059 (5)	−0.0005 (5)
C5	0.0156 (6)	0.0164 (6)	0.0140 (6)	−0.0020 (5)	0.0065 (5)	−0.0020 (5)
C6	0.0187 (7)	0.0178 (6)	0.0135 (6)	−0.0011 (5)	0.0058 (5)	−0.0016 (5)
C7	0.0231 (7)	0.0202 (7)	0.0173 (7)	−0.0036 (6)	0.0084 (6)	0.0021 (5)
C8	0.0196 (7)	0.0222 (7)	0.0195 (7)	−0.0046 (6)	0.0099 (6)	−0.0007 (5)
C9	0.0146 (7)	0.0228 (7)	0.0179 (7)	−0.0016 (5)	0.0058 (5)	0.0004 (5)
C10	0.0169 (7)	0.0162 (6)	0.0146 (6)	−0.0012 (5)	0.0070 (5)	−0.0014 (5)
C11	0.0274 (8)	0.0308 (8)	0.0247 (8)	−0.0053 (6)	0.0156 (7)	−0.0049 (6)
C12	0.0241 (8)	0.0297 (8)	0.0277 (8)	−0.0080 (6)	0.0137 (7)	0.0003 (6)
C13	0.0268 (8)	0.0236 (7)	0.0180 (7)	−0.0033 (6)	0.0061 (6)	0.0033 (6)
C14	0.0225 (7)	0.0266 (7)	0.0166 (7)	−0.0058 (6)	0.0092 (6)	−0.0055 (6)
C15	0.0261 (8)	0.0496 (11)	0.0316 (9)	−0.0113 (8)	0.0174 (7)	−0.0048 (8)
C16	0.0397 (11)	0.0598 (13)	0.0417 (11)	−0.0258 (10)	0.0186 (9)	−0.0104 (10)
C17	0.0157 (6)	0.0156 (6)	0.0140 (6)	−0.0022 (5)	0.0067 (5)	0.0004 (5)
C18	0.0168 (6)	0.0162 (6)	0.0141 (6)	−0.0022 (5)	0.0083 (5)	0.0013 (5)
C19	0.0172 (7)	0.0182 (6)	0.0164 (7)	−0.0014 (5)	0.0076 (5)	0.0013 (5)
C20	0.0200 (7)	0.0205 (7)	0.0220 (7)	0.0022 (5)	0.0115 (6)	0.0026 (5)
C21	0.0301 (8)	0.0174 (7)	0.0253 (8)	0.0001 (6)	0.0175 (7)	−0.0008 (6)
C22	0.0260 (8)	0.0188 (7)	0.0199 (7)	−0.0070 (6)	0.0125 (6)	−0.0046 (5)
C23	0.0207 (7)	0.0170 (6)	0.0162 (7)	−0.0041 (5)	0.0094 (6)	0.0005 (5)
C24	0.0207 (7)	0.0198 (7)	0.0152 (6)	−0.0056 (5)	0.0057 (6)	−0.0006 (5)
C25	0.0166 (7)	0.0189 (7)	0.0169 (7)	−0.0043 (5)	0.0062 (5)	0.0029 (5)
C26	0.0154 (6)	0.0170 (6)	0.0168 (6)	−0.0027 (5)	0.0074 (5)	0.0023 (5)
C27	0.0176 (7)	0.0195 (7)	0.0209 (7)	−0.0006 (5)	0.0087 (6)	0.0014 (5)
C28	0.0193 (7)	0.0220 (7)	0.0275 (8)	0.0017 (6)	0.0125 (6)	0.0055 (6)
C29	0.0144 (7)	0.0262 (7)	0.0260 (8)	−0.0014 (6)	0.0067 (6)	0.0094 (6)
C30	0.0175 (7)	0.0230 (7)	0.0193 (7)	−0.0060 (5)	0.0046 (6)	0.0041 (6)

### Ethyl 4-(anthracen-9-yl)-2,7,7-trimethyl-5-oxo-1,4,5,6,7,8-hexahydroquinoline-3-carboxylate (III) Geometric parameters (Å, º)

**Table:**

O1—C6	1.2370 (17)	C9—C10	1.5036 (19)
O2—C14	1.2138 (19)	C15—C16	1.510 (3)
O3—C14	1.3489 (19)	C17—C18	1.4188 (19)
O3—C15	1.4486 (19)	C17—C26	1.4188 (19)
N1—C2	1.3893 (18)	C18—C19	1.4342 (19)
N1—C10	1.3658 (18)	C18—C23	1.4377 (19)
C2—C3	1.350 (2)	C19—C20	1.362 (2)
C2—C13	1.5013 (19)	C20—C21	1.416 (2)
C3—C4	1.5275 (18)	C21—C22	1.356 (2)
C3—C14	1.4749 (19)	C22—C23	1.430 (2)
C4—C5	1.5220 (18)	C23—C24	1.391 (2)
C4—C17	1.5413 (18)	C24—C25	1.391 (2)
C5—C6	1.4430 (19)	C25—C26	1.4417 (19)
C5—C10	1.3550 (19)	C25—C30	1.430 (2)
C6—C7	1.5146 (19)	C26—C27	1.4356 (19)
C7—C8	1.527 (2)	C27—C28	1.364 (2)
C8—C9	1.534 (2)	C28—C29	1.419 (2)
C8—C11	1.534 (2)	C29—C30	1.358 (2)
C8—C12	1.533 (2)		
			
C14—O3—C15	116.95 (13)	O2—C14—O3	122.01 (14)
C10—N1—C2	122.41 (12)	O2—C14—C3	126.59 (14)
N1—C2—C13	112.76 (12)	O3—C14—C3	111.41 (12)
C3—C2—N1	119.95 (12)	O3—C15—C16	111.51 (14)
C3—C2—C13	127.23 (13)	C18—C17—C4	120.66 (12)
C2—C3—C4	122.61 (12)	C18—C17—C26	119.36 (12)
C2—C3—C14	119.20 (13)	C26—C17—C4	119.90 (12)
C14—C3—C4	117.97 (12)	C17—C18—C19	123.51 (12)
C3—C4—C17	112.38 (11)	C17—C18—C23	119.94 (12)
C5—C4—C3	110.73 (11)	C19—C18—C23	116.53 (12)
C5—C4—C17	114.41 (11)	C20—C19—C18	121.93 (13)
C6—C5—C4	118.40 (12)	C19—C20—C21	120.87 (14)
C10—C5—C4	122.21 (12)	C22—C21—C20	119.68 (13)
C10—C5—C6	118.85 (12)	C21—C22—C23	121.31 (13)
O1—C6—C5	121.10 (13)	C22—C23—C18	119.67 (13)
O1—C6—C7	119.63 (12)	C24—C23—C18	119.61 (13)
C5—C6—C7	119.20 (12)	C24—C23—C22	120.69 (13)
C6—C7—C8	115.17 (12)	C25—C24—C23	121.68 (13)
C7—C8—C9	107.55 (11)	C24—C25—C26	119.48 (13)
C7—C8—C11	110.50 (12)	C24—C25—C30	120.32 (13)
C7—C8—C12	109.77 (12)	C30—C25—C26	120.17 (13)
C9—C8—C11	110.82 (12)	C17—C26—C25	119.88 (12)
C12—C8—C9	108.59 (12)	C17—C26—C27	124.04 (13)
C12—C8—C11	109.57 (12)	C27—C26—C25	116.07 (12)
C10—C9—C8	112.84 (11)	C28—C27—C26	121.87 (14)
N1—C10—C9	115.33 (12)	C27—C28—C29	121.26 (14)
C5—C10—N1	120.82 (12)	C30—C29—C28	119.33 (13)
C5—C10—C9	123.84 (12)	C29—C30—C25	121.17 (14)
			
O1—C6—C7—C8	−160.55 (13)	C13—C2—C3—C4	175.60 (13)
N1—C2—C3—C4	−1.6 (2)	C13—C2—C3—C14	1.1 (2)
N1—C2—C3—C14	−176.08 (12)	C14—O3—C15—C16	−82.03 (19)
C2—N1—C10—C5	−10.5 (2)	C14—C3—C4—C5	167.62 (12)
C2—N1—C10—C9	168.21 (12)	C14—C3—C4—C17	−63.06 (16)
C2—C3—C4—C5	−6.93 (18)	C15—O3—C14—O2	−7.7 (2)
C2—C3—C4—C17	122.39 (14)	C15—O3—C14—C3	172.08 (12)
C2—C3—C14—O2	−25.1 (2)	C17—C4—C5—C6	67.85 (16)
C2—C3—C14—O3	155.09 (13)	C17—C4—C5—C10	−120.75 (14)
C3—C4—C5—C6	−163.92 (11)	C17—C18—C19—C20	178.82 (13)
C3—C4—C5—C10	7.48 (18)	C17—C18—C23—C22	−179.25 (12)
C3—C4—C17—C18	−65.27 (16)	C17—C18—C23—C24	−1.38 (19)
C3—C4—C17—C26	111.42 (14)	C17—C26—C27—C28	−175.04 (13)
C4—C3—C14—O2	160.16 (14)	C18—C17—C26—C25	1.94 (19)
C4—C3—C14—O3	−19.65 (17)	C18—C17—C26—C27	−179.50 (12)
C4—C5—C6—O1	2.40 (19)	C18—C19—C20—C21	0.1 (2)
C4—C5—C6—C7	179.41 (12)	C18—C23—C24—C25	0.8 (2)
C4—C5—C10—N1	0.6 (2)	C19—C18—C23—C22	−0.43 (18)
C4—C5—C10—C9	−178.06 (12)	C19—C18—C23—C24	177.45 (12)
C4—C17—C18—C19	−2.05 (19)	C19—C20—C21—C22	0.2 (2)
C4—C17—C18—C23	176.69 (12)	C20—C21—C22—C23	−0.6 (2)
C4—C17—C26—C25	−174.79 (12)	C21—C22—C23—C18	0.8 (2)
C4—C17—C26—C27	3.8 (2)	C21—C22—C23—C24	−177.10 (13)
C5—C4—C17—C18	62.12 (16)	C22—C23—C24—C25	178.66 (13)
C5—C4—C17—C26	−121.19 (13)	C23—C18—C19—C20	0.04 (19)
C5—C6—C7—C8	22.39 (18)	C23—C24—C25—C26	1.1 (2)
C6—C5—C10—N1	171.93 (12)	C23—C24—C25—C30	−177.01 (13)
C6—C5—C10—C9	−6.7 (2)	C24—C25—C26—C17	−2.52 (19)
C6—C7—C8—C9	−49.79 (16)	C24—C25—C26—C27	178.81 (12)
C6—C7—C8—C11	71.30 (15)	C24—C25—C30—C29	178.32 (13)
C6—C7—C8—C12	−167.75 (12)	C25—C26—C27—C28	3.6 (2)
C7—C8—C9—C10	49.90 (15)	C26—C17—C18—C19	−178.75 (12)
C8—C9—C10—N1	157.27 (12)	C26—C17—C18—C23	−0.01 (19)
C8—C9—C10—C5	−24.03 (19)	C26—C25—C30—C29	0.2 (2)
C10—N1—C2—C3	11.0 (2)	C26—C27—C28—C29	−1.2 (2)
C10—N1—C2—C13	−166.55 (13)	C27—C28—C29—C30	−1.8 (2)
C10—C5—C6—O1	−169.29 (13)	C28—C29—C30—C25	2.3 (2)
C10—C5—C6—C7	7.72 (19)	C30—C25—C26—C17	175.63 (12)
C11—C8—C9—C10	−70.98 (15)	C30—C25—C26—C27	−3.04 (19)
C12—C8—C9—C10	168.62 (12)		

### Ethyl 4-(anthracen-9-yl)-2,7,7-trimethyl-5-oxo-1,4,5,6,7,8-hexahydroquinoline-3-carboxylate (III) Hydrogen-bond geometry (Å, º)

**Table:**

*D*—H···*A*	*D*—H	H···*A*	*D*···*A*	*D*—H···*A*
N1—H1···O1^i^	0.90 (2)	1.94 (2)	2.7776 (16)	154.2 (18)
C13—H13*B*···O2^ii^	0.98	2.65	3.409 (2)	134
C19—H19···N1	0.95	2.48	3.4148 (19)	168

Symmetry codes: (i) *x*+1/2, −*y*+3/2, *z*+1/2; (ii) −*x*+1, −*y*+1, −*z*+1.
